# Complex mitogenomic rearrangements within the Pectinidae (Mollusca: Bivalvia)

**DOI:** 10.1186/s12862-022-01976-0

**Published:** 2022-03-10

**Authors:** Tamás Malkócs, Amélia Viricel, Vanessa Becquet, Louise Evin, Emmanuel Dubillot, Eric Pante

**Affiliations:** 1 Littoral Environnement et Sociétés (LIENSs), UMR 7266, CNRS-Université de La Rochelle, 2 Rue Olympe de Gouges, 17042 La Rochelle Cedex 01, France; 2 Pál Juhász-Nagy Doctoral School of Biology and Environmental Sciences, University of Debrecen, Egyetem tér 1, 4032 Debrecen, Hungary; 3 Institute of Biology and Ecology, University of Debrecen, Egyetem tér 1, 4032 Debrecen, Hungary; 4 Institute of Aquatic Ecology, Centre for Ecological Research, 4026 Debrecen, Hungary

**Keywords:** Mitogenome, Gene rearrangement, Pectinidae, De novo assembly, Variegated scallop, RNA-seq

## Abstract

**Background:**

Scallops (Bivalvia: Pectinidae) present extraordinary variance in both mitochondrial genome size, structure and content, even when compared to the extreme diversity documented within Mollusca and Bivalvia. In pectinids, mitogenome rearrangements involve protein coding and rRNA genes along with tRNAs, and different genome organization patterns can be observed even at the level of Tribes. Existing pectinid phylogenies fail to resolve some relationships in the family, Chlamydinae being an especially problematic group.

**Results:**

In our study, we sequenced, annotated and characterized the mitochondrial genome of a member of Chlamydinae, *Mimachlamys varia—*a species of commercial interest and an effective bioindicator—revealing yet another novel gene arrangement in the Pectinidae. The phylogeny based on all mitochondrial protein coding and rRNA genes suggests the paraphyly of the *Mimachlamys* genus, further commending the taxonomic revision of the classification within the Chlamydinae subfamily. At the scale of the Pectinidae, we found that 15 sequence blocks are involved in mitogenome rearrangements, which behave as separate units.

**Conclusions:**

Our study reveals incongruities between phylogenies based on mitochondrial protein-coding versus rRNA genes within the Pectinidae, suggesting that locus sampling affects phylogenetic inference at the scale of the family. We also conclude that the available taxon sampling does not allow for understanding of the mechanisms responsible for the high variability of mitogenome architecture observed in the Pectinidae, and that unraveling these processes will require denser taxon sampling.

**Supplementary Information:**

The online version contains supplementary material available at 10.1186/s12862-022-01976-0.

## Background

Historically, animal mitochondrial genomes were thought to have a conserved size of approximately 16 kbp, as well as a conserved structure, containing 13 protein coding genes (PCGs), two rRNA genes, and 22 tRNA genes and a single non-coding, ‘control region’ that regulates replication and transcription [1, 2]. In contrast, molluscan mitochondrial genomes provide examples of exceptional variation in size, structure and even function. Mitogenome size in Mollusca ranges from ~ 14 kbp up to 56.2 kbp [3–7], gene rearrangements, duplications, losses and inversions are frequent e.g. [8], and there is evidence of the integration of novel genes e.g. [9]. Additionally, a unique, complex mitochondrial inheritance system, called ‘doubly uniparental inheritance’ evolved in gonochoric bivalve mollusks [10–12].

Regarding mitogenome structure, scallops (Bivalvia: Pectinidae) constitute one of the most peculiar groups within Mollusca. They include one of the largest mitogenomes known (32–42 kbp; [7], with other species having mitogenomes of different lengths ranging from 16 to 21 kbp [13, 14]. They also present the most variation in mitochondrial gene order, even at relatively shallow phylogenetic depths, i.e. within subfamilies [14]. In Pectinidae, as well as among metazoans in general, gene rearrangements are most common for tRNAs [14–17]. Several pectinids contain extra tRNAs beyond the essential 22 needed for mitochondrial translation, either duplicated genes, or genes encoding for the same amino acid, but using a different anticodon [18–21]. However, even more species contain fewer than 22 mitochondrial tRNAs [13, 14, 19, 22, 23], possibly coming from annotation errors, which might be the result of modified tRNA secondary structure, common in other mollusks [24–26]. Similarly, *atp8* genes were reported earlier as missing from most bivalves, including pectinids [13, 14, 18, 20], although reannotation efforts revealed its presence in several species [15, 21, 27]. The difficulty of annotating the *atp8* gene is thought to stem from the poorly conserved sequence of this gene in bivalves [28–30]. Unlike what was observed in other animal groups, mitochondrial genome rearrangements in Pectinidae often involve PCGs [13–15]. This, however, does not seem to be correlated to sequence similarities among PCGs [14]. Furthermore, like most bivalves, most pectinid mitochondrial genomes contain several inflated non-coding regions, some of which are hypothesized to function as control regions [13, 14, 18, 20], although it remains controversial whether several control regions within a mitogenome could provide normal function [20].

Most existing pectinid phylogenies are based on one, or a handful of gene sequences, of either just mitochondrial, or of both mitochondrial and nuclear origin [31–41]. These phylogenies are often incongruent with morphological classifications of the Pectinidae [40]. Also, while they conclude that the Pectinidae is monophyletic, many lower taxonomic levels are reported as being paraphyletic [31, 40]. Phylogenies based on full mitogenomes have shown their utility in disentangling evolutionary relationships that were controversial when investigated using only a few genes [16, 42–44]. There are a few examples of pectinid phylogenies based on complete mitogenomes [14, 15, 18, 22, 27], however, most of these studies rely solely on genetic distance-based methods (except: [15, 22]), without considering the differences in substitution rates among different mitochondrial genes. Despite the diversity of approaches, some phylogenetic relationships remain to be resolved, for example, those within the Chlamydinae subfamily.

Numerous scallop species are harvested for human consumption and are used as environmental indicators. One example is the variegated scallop (*Mimachlamys varia* L., 1758), a species that inhabits the subtidal zone of the European Atlantic and the Mediterranean Sea [45]. It was demonstrated that *Mim. varia* is a potent bioindicator, as it presents significant physiological responses to chemical contaminants, including changes in biomarkers connected to oxidative stress, immune system function and mitochondrial respiration [46–48]. Most existing genetic research involving *Mim. varia* focused on the use of a few genes for phylogenetic or population genetic inference [31, 40, 49]. Viricel et al. [50] published the transcriptome of *Mim. varia*, providing a valuable resource for further ecophysiological and ecotoxicological research, including analyses of differential gene expression in response to marine pollution and comparative genomic studies within the Pectinidae.

In this study, our goals were to: (I) assemble and characterize the mitochondrial genome of the variegated scallop (*Mimachlamys varia*); (II) reconstruct the most up to date pectinid phylogeny using all available mitochondrial genomes, including *Mim. varia*; (III) perform comparative analyses of pectinid mitochondrial gene orders, to investigate the evolution of mitogenome arrangements within this family.

## Results

### Sequencing of the mitogenome of reference for the variegated scallop

**Fig. 1:**
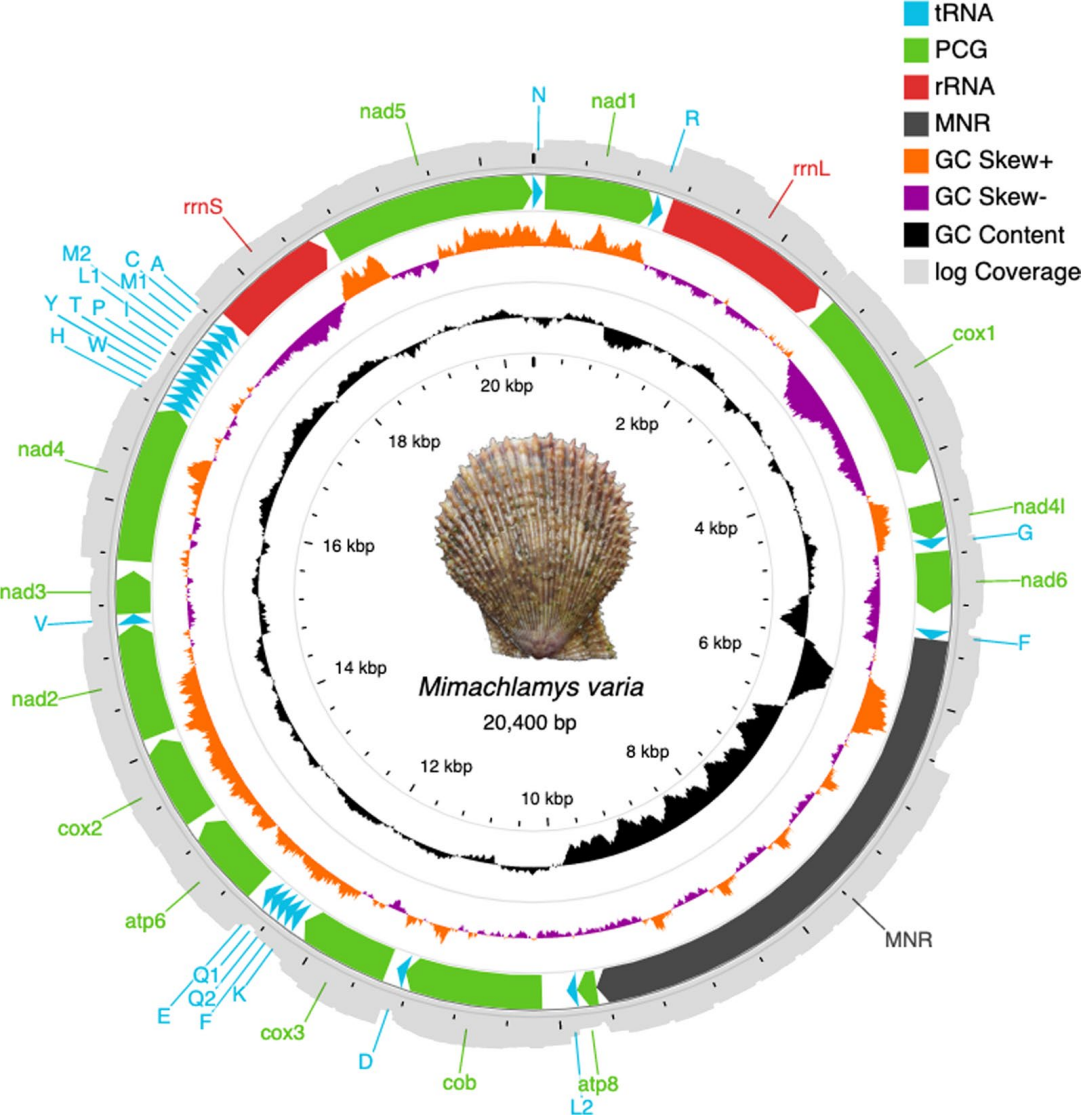
Map of the *Mimachlamys varia* mitogenome. Protein coding (CDS), rRNA and tRNA genes and the Major Non-coding Region (MNR) are marked with differently colored arrows pointing in the direction of transcription. Innermost scale represents size in kbp. GC content and GC skew are represented by black and purple-orange histograms, respectively. The outermost circle in light gray shows the natural logarithm of the depth of coverage for each base. Photo credit: Thierry Guyot

The NOVOPlasty assembly, based on a single *cox1* seed, yielded four contigs, 5289, 530, 3458 and 13,528 bp long, recruiting about 5.9 M reads (9.67% of the 61.1 M RNAseq reads submitted to the assembler), with an average depth of coverage of 84,143. NOVOPlasty never retrieved a full, circular mitogenome based on these data; the final assembly is based on the manual assembly of the four, overlapping contigs in Geneious, resulting in a complete, 20,400 bp long circular assembly. In total, 6.9 M reads were remapped onto the assembled genome. After remapping, the breath of coverage is 99.98%, only 5 bases are not covered around the site where the circular genome was split into a linear sequence. Coverage depth values ranged from 11–4,160,626 (Fig. 1). The mean and median coverage depths were 100,437 and 14,687, respectively.

#### Genome composition

The full mitogenome contains 13 Protein Coding Genes (PCGs; including an *atp8*, reported as missing for many bivalves; [13, 14, 18, 20]), 23 tRNAs, and 2 rRNAs (Fig. 1, Table 1). All genes are coded on the “+” strand. Two 2 bp overlaps were detected by MITOS2, between *nad1–trnR* and *cob–trnD*. Global A+T content is 56.3%. No heteroplasmy was detected with the two MAF settings used in NOVOPlasty.

**Table 1:** Main structural features of the *Mimachlamys varia* mitogenome

Gene	Location	Size (bp)	Start codon	Stop codon	Anticodon	Intergenic nucleotides	No. AA	A+T content (%)
trnN	1–69	69			GUU	–		54
nad1	94–993	900	TTG	TAG		24	299	56.1
trnR	992–1055	64			UCG	− 2		56.2
rrnL	1111–2532	1422				55		56.6
cox1	2604–4127	1524	ATG	TAG		71	507	55
nad4l	4384–4671	288	TTG	TAG		256	95	50.3
trnG	4677–4739	63			UCC	5		60.3
nad6	4774–5271	498	ATT	TAA		34	165	54.6
trnF	5414–5483	70			AAA	142		58.6
atp8	9690–9848	159	ATG	TAG		4206	52	55.3
trnL2	9859–9924	66			UAA	10		50
cob	10,133–11,257	1125	ATG	TAG		208	374	56
trnD	11,256–11,321	66			GUC	− 2		47
cox3	11,401–12,171	771	TTG	TAA		79	256	55.5
trnK	12,215–12,283	69			UUU	43		46.4
trnF	12,288–12,352	65			GAA	4		49.2
trnQ2	12,359–12,425	67			UUG	6		52.2
trnQ1	12,429–12,497	69			UUG	3		44.1
trnE	12,503–12,568	66			UUC	5		54.5
atp6	12,642–13,313	672	ATG	TAA		73	223	56.8
cox2	13,377–14,078	702	ATG	TAG		63	233	54.7
nad2	14,084–15,040	957	ATA	TAA		41	306	56
trnV	15,051–15,116	66			UAC	10		50
nad3	15,125–15,472	348	ATT	TAG		8	115	54.9
nad4	15,552–16,835	1284	ATG	TAG		79	427	54.8
trnH	16,837–16,902	66			GUG	1		56.9
trnW	16,908–16,977	70			CCA	5		54.3
trnY	16,981–17,045	65			GUA	3		49.2
trnT	17,050–17,112	63			UGU	4		57.1
trnP	17,116–17,183	68			UGG	3		48.5
trnI	17,186–17,256	71			GAU	2		57.7
trnL1	17,265–17,333	69			UAG	8		53.6
trnM2	17,336–17,402	67			CAU	2		47.8
trnM1	17,411–17,483	73			CAU	8		41.1
trnC	17,492–17,559	68			GCA	8		42.6
trnA	17,571–17,638	68			UGC	11		59.1
rrnS	17,675–18,638	964				36		52.7
nad5	18,690–20,393	1704	ATT	TAA		51	567	55.9

#### Protein-coding genes

All 13 PCGs commonly occurring in metazoan mitochondrial genomes were identified in *Mim. varia*. The combined length of PCGs was 10,896 bp (45.4% of the complete genome). The total amino acid (AA) length was 3619, excluding stop codons. The most frequently used AAs were Leucine (12.86%), Valine (10.39%), Phenylalanine (9.55%), Glycine (9.45%) and Serine (8.31%). Six genes (*cox1, atp8, cob, atp6, cox2, nad4*) had the most common start codon ATG, three had ATT (*nad6, nad3, nad5*), three TTG (*nad1, nad4l, cox3*) and *nad2* had the ATA start codon (Table 1). Eight genes had the TAG and five had the TAA stop codon.

#### rRNA and tRNA genes

The *rrnS* and *rrnL* genes were 964 bp and 1422 bp long, respectively. Out of the total 23 tRNAs identified, most were identified by MITOS2, except *trnQ2*, which was identified by ARWEN. The length of tRNAs ranges from 63 to 73 nt. Three tRNA genes are present in two copies, *trnL* with different anticodons (*trnL1*: UAG, *trnL2*: UAA), *trnM* and *trnQ* with the same anticodon in both copies, CAU and UUG, respectively. All tRNAs were predicted to have the typical cloverleaf structure, except trnQ2 (Additional file 1). While present in two copies in most animal mitogenomes, *trnS* was not identified in the mitogenome of *Mim. varia*.

#### Gene order

The *Mim. varia* mitogenome represents a novel gene order for the Pectinidae. The conserved pectinid gene clusters “*nad1-trnR-rrnL-cox1*” and “*nad4-trnH-trnW*” are present, however, the “*nad6-trnL2-cob*” cluster is split by the insertion of *trnF*, the MNR and *atp8*, and the “*cob-cox2*” cluster is also missing. Notable is the relocation of the “*rrnS-nad5*” pair, and the location of the MNR between the *nad6* and *cob* genes, a pattern observed in only one other species, *Pl. magellanicus*.

#### Structure of the MNR

The major non-coding region (MNR) spans 4206 nt and has an elevated A+T content of 60.1%. There were four repeated regions, with repeat sizes between 10 and 683 bp (Table 2). The largest repeat region contained 4.7 copies of a 683 bp long sequence, with a total length of 3205 bp, making up 76.2% of the MNR. We PCR-amplified the MNR but failed to obtain its complete sequence using Sanger sequencing, possibly because of numerous stem-and loop structures and high %AT in this region (Additional file 2).

**Table 2:** Tandem repeats in the *Mimachlamys varia* mitogenome

Position in genome	Period size	Copy number	Consensus size	Percent matches	Percent indels	Alignment score	A	C	G	T	Entropy (0–2)
5601–5625	13	1.9	13	100	0	50	84	0	16	0	0.63
5894–5935	10	4.3	10	87	6	68	21	28	40	9	1.84
5896–5933	19	2	20	94	5	69	21	26	42	10	1.85
6440–9644	683	4.7	683	99	0	6392	28	13	22	35	1.92

### Annotation of pectinid genomes

**Fig. 2:**
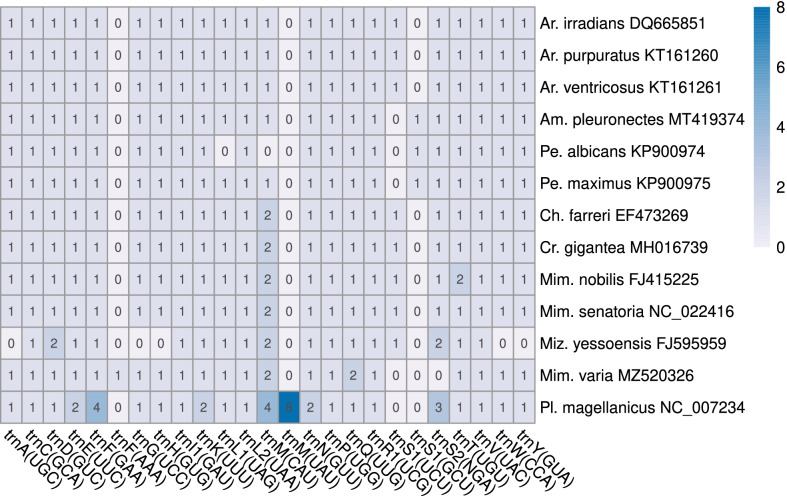
Heatmap representing mitochondrial tRNA gene sets observed in the 13 pectinid species included in this study. tRNAs are represented with their one-letter amino-acid code, with the anticodon in parentheses. The deeper blue color corresponds to higher copy number of a tRNA gene

Out of the 29 Pectinidae and 1 outgroup (*O. lurida*) mitogenomes retrieved from Genbank, only two were incomplete. All protein coding and rRNA genes identified in previous descriptions of these genomes were found and reannotated in this study. The *atp8* gene was annotated in all 25 genomes, in which it was not previously annotated, including two paralogous copies in *Mizuhopecten yessoensis* and *Pl. magellanicus*, and unusually long versions of the gene in two *Ar. irradians* GenBank accessions—with alternative start codons—showing only 78.5% sequence similarity with conspecific *atp8* genes (Table 3, Additional file 3). Between 20 and 40 tRNA genes were annotated per mitogenome in the 29 pectinid species. Most mitogenomes contained 23 tRNA genes, compared to the 22 commonly found in animals. Gene content was mainly identical within species, but there are slight variations, some of which bears a phylogenetic pattern. For example, the trnS1 genes in *Amusium pleuronectes*, *Pecten albicans* and *Pecten maximus* all have GCU as anticodon, and all species in the Chlamydinae subfamily contain two copies of the trnM gene. Some variations are present in only one species, *Pe. albicans* missing trnL1 and trnM, *Mimachlamys nobilis* containing an extra trnT gene, *Miz. yessoensis* lacking five tRNA genes, while containing two supernumerary ones, and *Pl. magellanicus* containing 12 trnM, 4 trnF and 3 trnS2 genes (Fig. 2), although most of these were identified as pseudogenes by Smith et al. [20].

**Table 3:** Newly annotated *atp8* genes

Species	GenBank accession no	Start	Stop	Length	AA	5ʹ neighbor	3ʹ neighbor
*Placopecten magellanicus*	DQ088274–*atp8*-1	23,484	23,660	177	58	trnM	atp6
	DQ088274–*atp8*-2	64	231	168	55	trnG	trnM
	NC_007234–*atp8*-1	23,484	23,660	177	58	trnM	atp6
	NC_007234–*atp8*-2	64	231	168	55	trnG	trnM
*Chlamys farreri*	EF473269	10,041	10,187	147	48	nad5	trnD
	EU715252	10,041	10,187	147	48	nad5	trnD
	NC_012138	10,041	10,187	147	48	nad5	trnD
*Mizuhopecten yessoensis*	AB271769–*atp8*-1	9797	9943	147	48	nad4l	trnD
	AB271769–*atp8*-2	9239	9385	147	48	nad5	nad4l
	FJ595959–*atp8*-1	9797	9943	147	48	nad4l	trnD
	FJ595959–*atp8*-2	9239	9385	147	48	nad5	nad4l
	NC_009081–*atp8*-1	9797	9943	147	48	nad4l	trnD
	NC_009081–*atp8*-2	9239	9385	147	48	nad5	nad4l
*Crassadoma gigantea*	MH016739	11,145	11,303	159	52	nad5	trnD
*Mimachlamys nobilis*	FJ415225	7938	8090	153	50	nad4l	trnM
	FJ595958	7937	8089	153	50	nad4l	trnM
	NC_011608	7938	8090	153	50	nad4l	trnM
*Mimachlamys varia*	MZ520326	9690	9848	159	52	trnF	trnL2
*Pecten albicans*	KP900974	6000	6134	135	44	trnE	nad6
*Pecten maximus*	KP900975	5993	6127	135	44	trnE	nad6
*Argopecten irradians*	DQ665851	4272	4406	135	44	trnE	nad6
	EU023915	4185	4406	222	73	trnF	nad6
	KT161259	4272	4406	135	44	trnE	nad6
	KT161262	4272	4406	135	44	trnE	nad6
	KU589290	4272	4406	135	44	trnE	nad6
	NC_009687	4185	4406	222	73	trnF	nad6
	NC_012977	4272	4406	135	44	trnE	nad6
*Argopecten purpuratus*	KF601246	4276	4410	135	44	trnE	nad6
	KT161260	4276	4410	135	44	trnE	nad6
	NC_027943	4276	4410	135	44	trnE	nad6
*Argopecten ventricosus*	KT161261	4238	4372	135	44	trnE	nad6

### Mito-phylogenomics

**Fig. 3:**
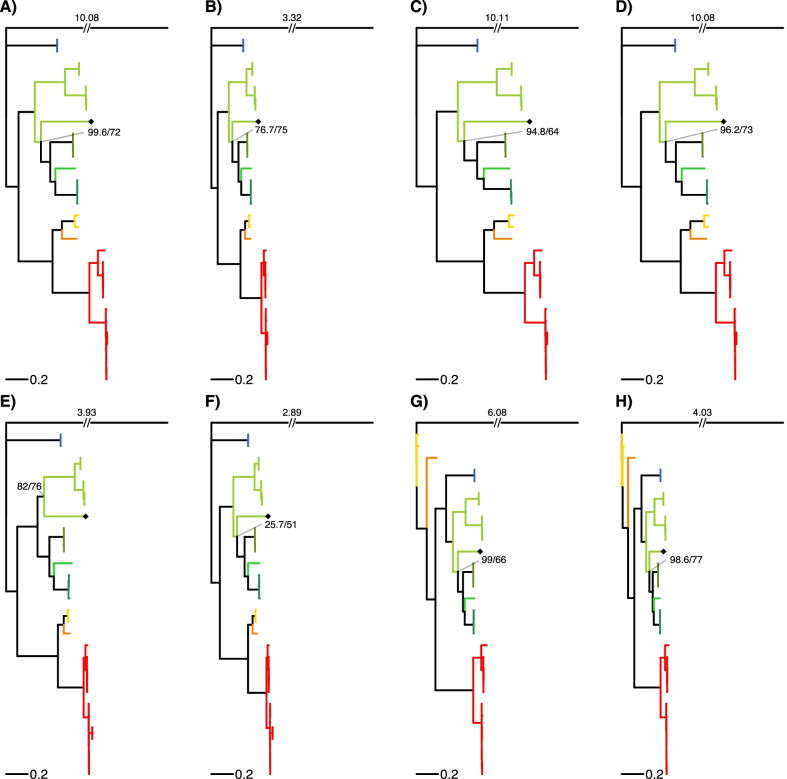
Maximum likelihood trees based on all eight datasets (**A**–**H**). Branch lengths are scaled by setting the length of the outgroup branch to 1.5 on each tree. Numbers on top of the outgroup branches represent the original length of the branches. Every genus is marked with a different color, which is identical to the color coding on Figs. 4 and 5. *Mimachlamys varia* is marked with a black diamond shape, the gray line connects the corresponding internal node with the SH-like approximate likelihood ratio test (SH-aLRT) value on the left and ultrafast bootstrap support on the right. Dataset descriptions: **A** all PCG and rRNA genes treated with Gblocks; **B** all PCG amino acid sequences and rRNA genes treated with Gblocks; **C** all PCGs; **D** all PCGs treated with Gblocks; **E** all PCG amino acid sequences; **F** all PCG amino acid sequences treated with Gblocks; **G** rRNA genes; **H** rRNA genes treated with Gblocks

**Fig. 4:**
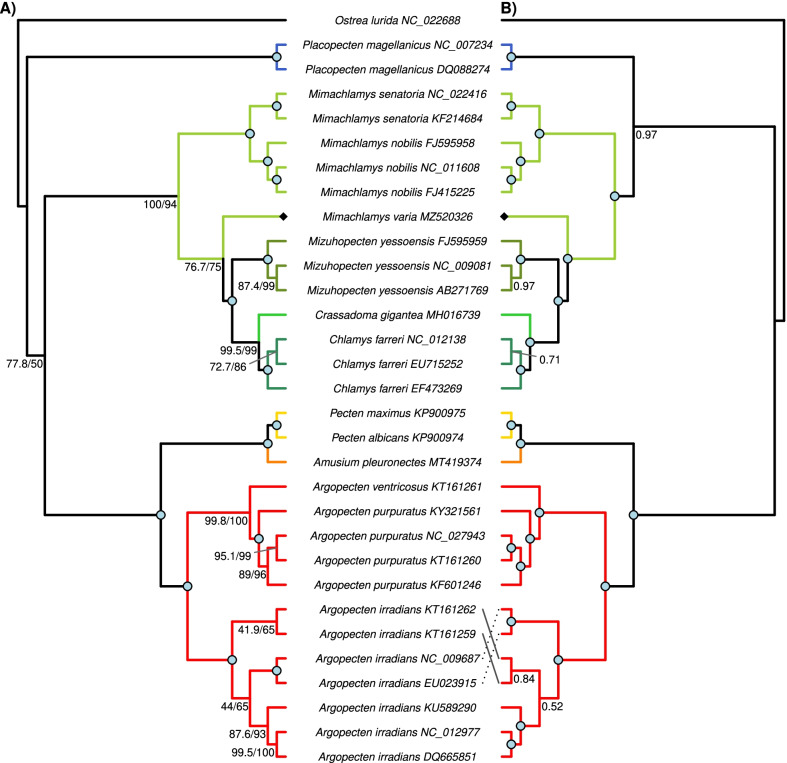
Phylogenetic reconstruction of 30 Pectinidae mitogenomes with *Ostrea lurida* (Ostreidae) as the outgroup, based on the “B” dataset (PCG amino acid and ribosomal DNA sequences). Every genus is marked with a different color, identical to the color coding on Figs. 3 and 5. *Mimachlamys varia* is marked with a black diamond shape. Fully supported branches are marked with a blue circle on the corresponding node. **A** Maximum likelihood tree. SH-like approximate likelihood ratio test (SH-aLRT) is presented on the left, ultrafast bootstrap support projected from the extended majority-rule consensus tree is presented on the right. **B** Bayesian tree. Numbers represent posterior probabilities; the staggered and gray lines connect species names with the corresponding tips. For original branch lengths, see Figs. 3B and 5B

All four subfamilies and 7 out of 9 tribes of the Pectinidae family are represented in this study (Table 4). Two different topologies can be observed among the ML trees reconstructed from the eight datasets (Fig. 3). The two rRNA datasets (G and H) show a distinct topology compared to the other 6 datasets, but the topology based on the rRNA datasets is not well-supported. All PCG ML trees had very similar topology. *Mim. varia* is placed within the same clade with the other two congeneric species only in the tree based on the E dataset (PCG amino acid sequences). In every other tree *Mim. varia* is placed in a clade with species in the Crassadomini and Chlamydini tribes. The monophyly of neither of these clades is supported. Similarly, although the topology suggests that *Pl. magellanicus* is sister to all other pectinids, its placement is never supported as such, rather the topology of the Pectinidae should be considered as a trichotomy represented by the subfamilies Palliolinae, Chlamydinae and a Pectininae + Aequipectini group. The B dataset resulted in the most well-supported tree (Fig. 4A). All eight ML trees presented with proportional branch lengths are shown in Fig. 3.

**Table 4:** List of species included in the present study

Subfamily	Tribe	Species	GenBank accession no	Genome length (bp)	Source
Palliolinae	Palliolini	*Placopecten magellanicus* (Gmelin, 1791)	DQ088274	32,115	La Roche et al. [77]⁠
			NC_007234	32,115	La Roche et al. [77]⁠
Chlamydinae	Chlamydini	*Chlamys farreri* (Jones & Preston, 1904)	EF473269	20,889	Ren et al. [11]⁠
			EU715252	21,695	Xu et al. [18]⁠
			NC_012138	21,695	Xu et al. [18]⁠
		*Mizuhopecten yessoensis* (Jay, 1857)	AB271769	20,414	Sato et al. [14]⁠
			FJ595959	20,964	Wu et al. [12]⁠
			NC_009081	20,414	Sato et al. [14]⁠
	Crassadomini	*Crassadoma gigantea* (J.E. Gray, 1825)	MH016739	18,495	Liao et al. [13]⁠
	Mimachlamydini	*Mimachlamys nobilis* (Reeve, 1852)	FJ415225	17,963	Xu et al., unpublished
			FJ595958	17,935	Wu et al. [12]⁠
			NC_011608	17,963	Xu et al., unpublished
		*Mimachlamys senatoria* (Gmelin, 1791)	KF214684	17,383	Wu et al. [16]⁠
			NC_022416	17,383	Wu et al. [16]⁠
		*Mimachlamys varia* (Linnaeus, 1758)	MZ520326	20,400	this study
Pectininae	Amusiini	*Amusium pleuronectes* (Linnaeus, 1758)	MT419374	18,044	Yao et al. [23]⁠
	Pectinini	*Pecten albicans* (Schröter, 1802)	KP900974	16,653	Marín et al. [17]⁠
		*Pecten maximus* (Linnaeus, 1758)	KP900975	17,252	Marín et al. [17]⁠
Aequipectini group	Aequipectini	*Argopecten irradians* (Lamarck, 1819)	DQ665851	16,211	Ren et al. [11]⁠
			EU023915	16,221	Petten and Snyder, unpublished
			KT161259	16,286	Li, unpublished
			KT161262	16,211	Li, unpublished
			KU589290	16,212	Liu, unpublished
			NC_009687	16,221	Petten and Snyder, unpublished
			NC_012977	16,211	Ren et al. [11]⁠
		*Argopecten purpuratus* (Lamarck, 1819)	KF601246	16,266	Marín et al. [17]⁠
			KT161260	16,270	Li, unpublished
			KY321561	16,224	Romero et al., unpublished
			NC_027943	16,270	Li, unpublished
		*Argopecten ventricosus* (G. B. Sowerby II, 1842)	KT161261	16,079	Li, unpublished
Outgroup					
Family	Ostreaidae	*Ostrea lurida* Carpenter, 1864	NC_022688	16,344	Xiao et al. [100]⁠

Taxonomic authorities according to the World Register of Marine Species (WoRMS Editorial Board, 2021). Annotation information can be found in Table 1 for *Mim. varia*, and Additional file 3 for all other species

**Fig. 5:**
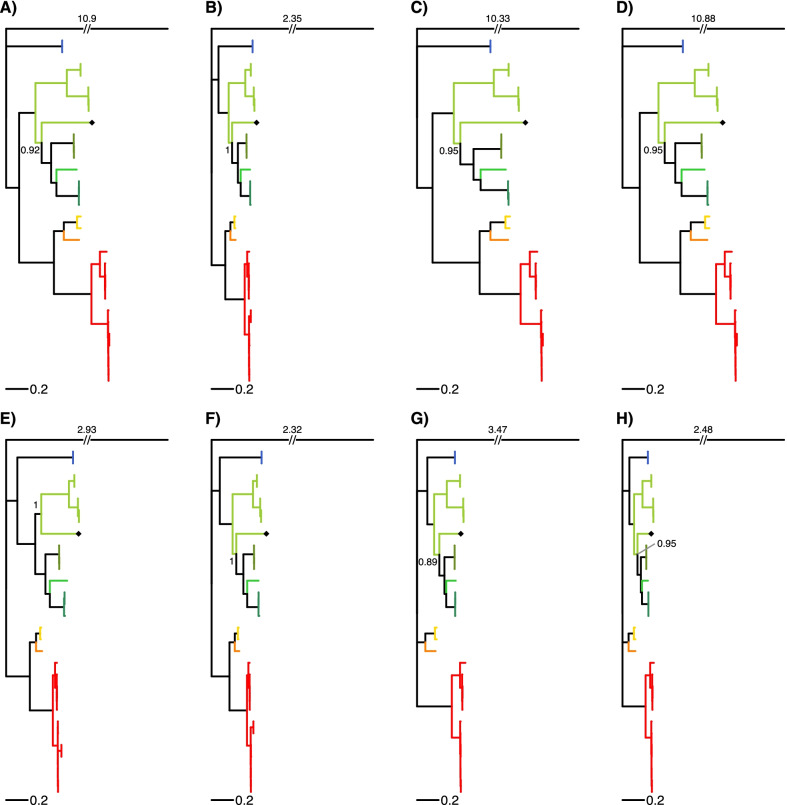
Bayesian consensus trees based on all eight datasets (**A**–**H**). Branch lengths are scaled by setting the length of the outgroup branch to 1.5 on each tree. Numbers on top of the outgroup branches represent the original length of the branches. Every genus is marked with a different color, that is identical to the color coding of Figs. 3 and 4. *Mimachlamys varia* is marked with a black diamond shape. Posterior probabilities of the corresponding internal node are presented. Dataset descriptions: **A** all PCG and rRNA genes masked with Gblocks; **B** all PCG amino acid sequences and rRNA genes masked with Gblocks; **C** all PCGs; **D** all PCGs masked with Gblocks; **E** all PCG amino acid sequences; **F** all PCG amino acid sequences masked with Gblocks; **G** rRNA genes; **H** rRNA genes masked with Gblocks

Every Bayesian phylogenetic reconstruction run on every dataset reached convergence (ESS > 200). The results of these analyses slightly differ from the results of the ML method (Fig. 4B). Support values are generally higher than in the ML approach. *Pl. magellanicus* is placed in the same clade with Chlamydinae in the trees B, E, F, G and H with moderate to high posterior probabilities (0.81–0.98). In the rRNA trees (G and H), there is a trichotomy at the root of the Pectinidae, where the three clades are formed by Palliolinae + Chlamydinae, Pectininae and Aequipectini groups, respectively. The placement of *Mim. varia* is identical to its placement in the ML trees. All eight Bayesian trees presented with proportional branch lengths are shown in Fig. 5.

### Genome rearrangements

#### Gene collinearity

**Fig. 6:**
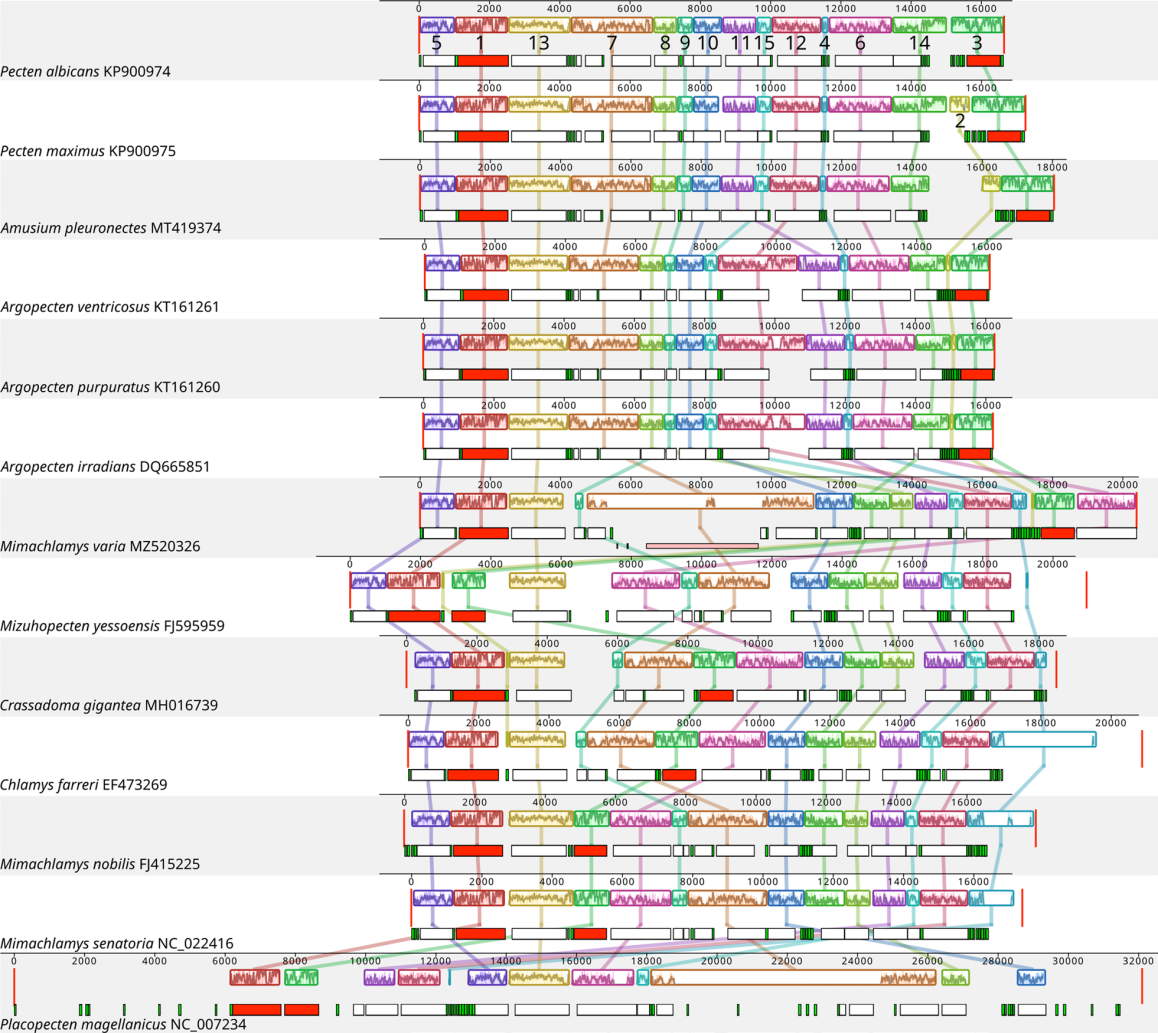
Gene collinearity analysis of 13 Pectinid species performed in Mauve v2.3.1. Color blocks represent homologous regions among different mitogenomes. The level of similarity at each position is shown in the blocks. Below these blocks, the white, red, green and pink boxes represent protein coding, rRNA, tRNA genes, and tandem repeats, respectively

**Fig. 7:**
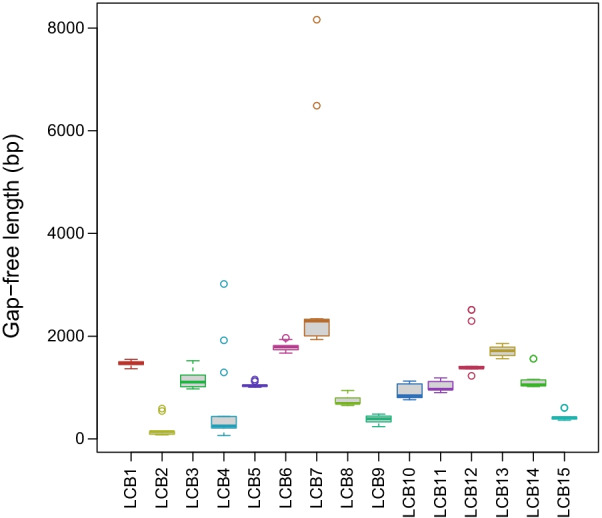
Boxplot showing the length distribution of each LCB (locally colinear block) from the gene collinearity analysis

Fifteen Locally Collinear Blocks were identified among 13 (1 per species) pectinid mitogenomes (Fig. 6). Most of these 15 blocks were present in all mitogenomes, except for LCB2 (missing from *Mim. nobilis*, *Mimachlamys senatoria*, *Pe. albicans* and *Pl. magellanicus*), LCB9 and LCB14 (both missing from *Pl. magellanicus*). Ten blocks contained one major gene (PCG or rRNA) and none or some tRNA genes. LCB6 and LCB9 contained one or two PCGs and none or some tRNA genes, LCB7 two to four PCGs and some tRNAs, while LCB2 and LCB4 both contained a set of tRNA genes only. LCBs 4, 7 and 12 were the most variable in terms of length (Fig. 7), due to the fact that these three blocks contained the Major Non-coding Region in 8 out of the 13 species (Fig. 6). The order of LCBs was generally more similar in more closely related species, and identical within *Argopecten*, between *Mim. nobilis* and *Mim. senatoria* and between *Chlamys farreri* and *Crassadoma gigantea*. *Mim. varia*, *Miz. yessoensis* and *Pl. magellanicus* presented substantially different genome organization from what could be expected by looking at their phylogenetic position alone. When we look at only a subset of the whole species list, several larger blocks can be observed. Larger blocks present in more than two species are the following: LCBs 5, 1 and 13 are present in the same order in 11 species (different in *Mim. yessoensis* and *Pl. magellanicus*); LCBs 5, 1, 13, 7, 8, 9 and 10 form a single block in the Pectininae and the three *Argopecten* species; LCBs 10, 14, 8, 11, 15, 12 and 4 form a block in the Chlamydinae.

#### Common interval analysis

**Fig. 8:**
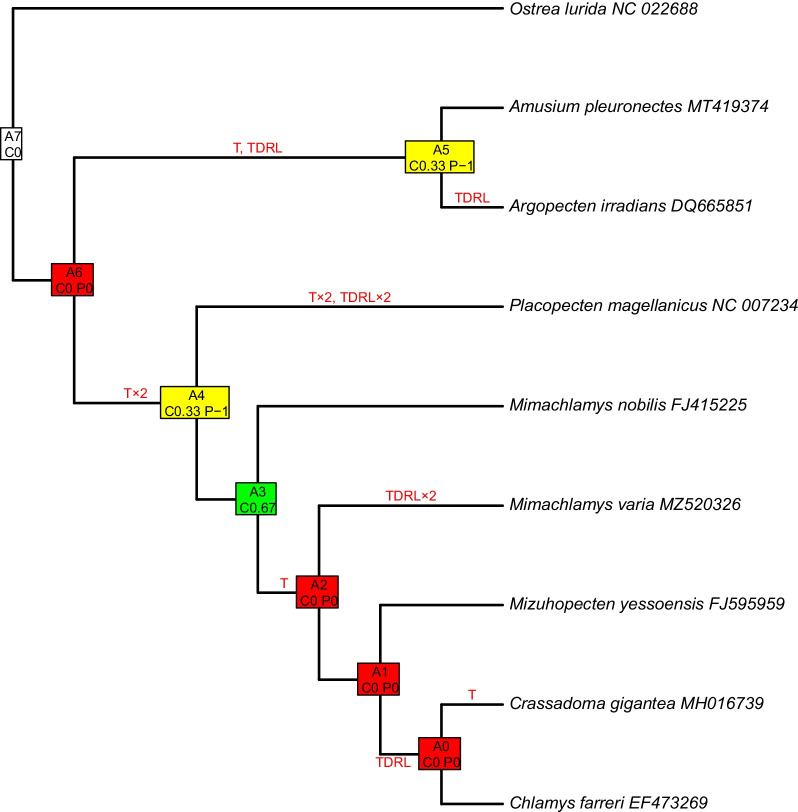
Common interval analysis presented on the most supported Bayesian tree, obtained from dataset “B”, where each tip represents a unique gene order. Internal nodes are named as “An”, where “n” is a number between 0 and 7, and represents ancestral gene orders. C and P indicate consistency and parsimony values, respectively. The colors code consistent (green; highest level of certainty), k-consistent (yellow; intermediate level of certainty) and inconsistent (red; low level of certainty) nodes. Mutation types are indicated on corresponding branches, T: transposition, TDRL: tandem duplication and random loss

Tandem-duplication/random loss and transposition events occurred seven times along the pectinid phylogeny (Fig. 8). Out of the 7 internal nodes, only one (the common ancestor of chlamydins) was consistent, with a high level of certainty, two were k-consistent, i.e., less reliable, with an intermediate level of certainty (the common ancestor of Chlamydinae + *Placopecten* and the common ancestor of Pectininae and *Argopecten*) and the rest were fallback nodes (representing the highest level of uncertainty), including the common ancestor of Pectinidae.

Three genes—*nad1*, *rrnL* and *cox1*—were involved in the fewest rearrangement events (Additional file 4).

#### Maximum likelihood analysis of gene orders

**Fig. 9:**
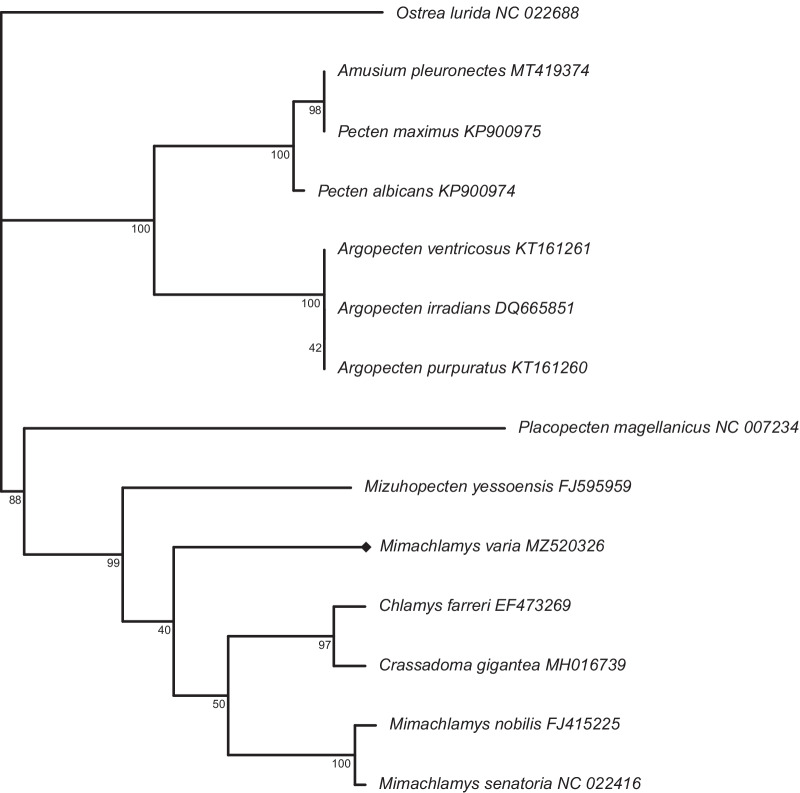
Maximum likelihood tree based on gene orders, 42 genes in total, including all 13 species included in this study plus the outgroup, and the complete set of genes in each mitogenome. Numbers represent bootstrap support. *Mimachlamys varia* is marked with a black diamond shape

In the maximum likelihood phylogeny reconstructed from gene orders, *Argopecten* is sister to pectinins, as in the eight ML and eight Bayesian trees, however, within Pectininae, *Am. pleuronectes* and *Pe. maximus* form the crown (Fig. 9). The other major differences are the positions of *Mim. varia* and *Miz. yessoensis,* the latter becoming the basal species in the Chlamydinae and *Mim. varia* becoming sister to the crown of the Chlamydinae, formed by the pairs of *Mim. nobilis*—*Mim. senatoria* and *Cr. gigantea*—*Ch. farreri* (Fig. 9).

## Discussion

To the best of our knowledge, our study presents the most complete mitochondrial comparative- and phylogenomic analysis to date in the Pectinidae family, including the newly-assembled mitogenome of *Mim. varia*. The composition of the latter conforms to most other pectinid mitogenomes published so far. However, it represents a completely novel gene order, previously not described in any other species of Pectinidae [13, 14, 18, 20–23, 27].

### Gene content

In addition to the 12 PCGs present in every bivalve mitogenomes, the *atp8* gene was annotated in *Mim. varia*. This is not surprising, given that *atp8* is being annotated in more and more newly published bivalve mitogenomes, as well as identified in mitogenomes where it was originally thought to be missing [15]. In our study, the *atp8* gene was annotated in 25 mitogenomes in which it was not previously found, in two species (*Miz. yessoensis*, *Pl. magellanicus*) it was even detected in two paralogous copies, and in two accessions of *Ar. irradians,* only as an unusual version of the gene (alternative start codon and a total length of 222 bp) (Table 3). The *atp8* gene is very short (135–222 bp in the Pectinidae), and is among the most variable mitochondrial PCGs, making it very difficult to detect. The discovery of a functional *atp8* gene in most pectinids hints at it not being accessory, as previously proposed [51–53]. Duplicated genes in mitochondrial genomes are not unheard of [54–56], although whether all copies are functional remains unknown.

While it is common for metazoan mitochondrial genomes to contain two trnL and trnS genes, pectinid mitogenomes also commonly contain two trnM genes. In accordance, the mitogenome of *Mim. varia* contains two trnL and trnM genes, however, it contains no trnS gene, and two trnF and trnQ genes. The two trnF genes show low sequence similarity and trnF (AAA) shows low sequence identity to trnF genes in other species as well as every tRNA gene in *Mim. varia*. Although with our methodology, trnQ2 was annotated in *Mim. varia*, this might be an artifact. We base this assumption on the fact that the two trnQ genes in *Mim. varia* are found next to each other, in the gene block (trnK, trnF, trnQ2, trnQ1, trnE), but the same gene block, containing trnS2 instead of trnQ2, is identified in *Mim. senatoria* [21] and *Cr. gigantea* [18], and revealed during reannotation in the current study in *Mim. nobilis*, *Ch. farreri* and *Miz. yessoensis* (Fig. 2, Additional file 3). Also, this trnQ2 gene shows higher sequence similarity to the trnS2 gene than to the trnQ gene in the aforementioned species. While we assume that this difference is the result of an artifact, it is not impossible that in *Mim. varia*, the trnS gene evolved into trnQ. This is supported by the fact that tRNA genes are often lost in mitochondrial genomes, and that remolding of tRNA anticodons is known to happen in mollusks [9, 21, 57].

### Structure of the MNR

High A+T content and stem-loop secondary structures are the common diagnostic traits in identifying the mitochondrial control region [58–60]. Both can be observed in the MNR of *Mim. varia* (Fig. 1, Additional file 2), pointing towards it serving as the control region.

### Annotation of pectinid genomes

The fact that we were able the annotate both the *atp8* gene and some new tRNA genes in most pectinid accessions downloaded for this study show that the lack of these genes were most likely artifacts emerging from the difficulty of annotating these features in bivalves. With the expansion of annotation databases and the advancement of annotation tools, these difficulties are getting easier and easier to overcome, leading to progressively more precise annotations.

Variations in tRNA gene content presented by singular species (especially those with only one mitogenome annotated: *Pe. albicans* and *Mim. varia*) might well be resulting from sequencing errors, incomplete genomes, or simply misannotations. While the possibility of these cannot be completely excluded, even in the case of species that have multiple genomes published (*Mim. nobilis, Miz. yessoensis, Pl. magellanicus*), those presenting a phylogenetic pattern are most probably real. A peculiar case is that of trnM, as it is present in more than half of all studied species (Fig. 2). This gene is present in two copies in several invertebrate mitogenomes [61, 62], including some bivalves [25]. The invertebrate mitochondrial genetic code contains five start codons, most of which code for amino acids other than methionine in vertebrate mitochondrial genomes. This means that trnM must do ‘double duty’ as the tRNA for both methionine and formyl-methionine, matching five instead of two start codons [58], which could explain why some invertebrates, including some pectinids evolved to have more copies of it in their mitochondrial genomes.

Numerous pectinid mitogenomes are longer than the usual metazoan mitochondrial genome (~ 16 kbp), for example *Aequipecten opercularis* 21–28.2 kbp, *Chlamys hastata* 23.9–27.2 kbp, *Chlamys islandica* 22.5–25 kbp, *Cr. gigantea* 22.8–24.8 kbp [63], *Mim. varia* 20.4 kbp (the current study), *Miz. yessoensis* 20.5–21 kbp [14], *Pl. magellanicus* 30.7–40.7 kbp [20], and *Pe. maximus* [22, 63, 64], although the available mitogenome for the latter species is only 17.2 kbp long. This is mostly due to inflated non-coding regions, as demonstrated by Ghiselli et al. [58] in the whole Mollusca phylum. We identified tandem repeats of total length larger than 500 bp in the non-coding regions of *Pl. magellanicus*, *Ch. farreri* (compare with [13]), *Mim. varia* and *Am. pleuronectes* (Additional file 5) and repeats of around 500 bp length scattered throughout the mitogenome of *Miz. yessoensis* (Additional file 3). Gjetvaj et al. [63] also found that most of the pectinid mitogenomes they studied, contain tandem repeats of various sizes and repeat numbers in their non-coding region, and they found no significant sequence similarity among these repeats. This supports the assumption that the repeats arose independently in every lineage. There is also apparent intraspecific variation in mitochondrial genome size within the Pectinidae [63–65].

While it is likely that correlation exist between the number and length of tandem repeats and genome size, the available number of pectinid mitogenomes does not allow to establish correlations between these traits.

The presence of multiple repeats make it seem very likely that tandem duplication is frequent in pectinid mitogenomes, perhaps enabled by the fact that all genes are coded on the “+” strand. However, this does not seem to influence the mitogenome architecture of other marine bivalves, most of which also code all genes on the “+” strand [13]. In plants, which usually have significantly larger mitogenomes than animals and contain numerous repeated sequences, it is suggested that these repeats promote recombination [66–68]. This might play a role in the evolution of the mitochondrial genome in the Pectinidae as well.

The prevalence of non-coding regions and, in some instances, extra tRNAs in Pectinidae make this group an interesting candidate to study the taxonomic distribution of small mitochondrial highly-transcribed RNAs (smithRNAs, [69]). This novel class of sncRNAs (short non-coding RNAs), initially described from the Manila clam *Ruditapes philippinarum* (class Venerida) and detected in zebrafish, fruitflies and mice [70], could influence the expression of nuclear-encoded genes and be involved in gonad formation. They were quasi-exclusively found in non-coding regions and tRNAs [69].

### Mito-phylogenomics

The phylogenies presented in our study are in accordance with previous results, both with those using a few genes, and those using the complete set of mitochondrial PCGs. One novel result is that in this group, the two rRNA genes seem to have little power to resolve deep divergences, as seen in the different placement of the Pectininae in both the ML and Bayesian approaches. In accordance with Puslednik et al. [40] and Alejandrino et al. [31], we found that *Mim. varia* is not placed into one monophyletic clade with the other two *Mimachlamys* species. Generally, although Chlamydinae itself is monophyletic, its lower taxonomic levels are not well-resolved, and several genera are paraphyletic, as it was presented in previous studies [22, 27, 31, 40, 71]. In contrast to Puslednik et al. [40], who concluded Aequipectini to be the basal clade in Pectinidae (similarly as shown in our rRNA datasets, G and H), we have found that Aequipectini always form a monophyletic group with Pectininae in both of our ML and Bayesian approaches, when we included PCGs in the analysis. It appears that while mitochondrial rRNA genes and the nuclear H3 gene are in accordance [40], mitochondrial PCGs paint a slightly different picture, as can also be seen in, for example, Marín et al. [22] and Yao et al. [27]. However, this could at least be partly caused by the small number of taxa involved in our study, and the different choice of outgroups, as both taxon sampling and outgroup selection are known to influence topologies [40]. Despite the Pectinidae being one of the largest families in Bivalvia, containing around 350 species [72], existing phylogenies usually contain only a handful of species, with the most making up only 31% of all species [31]. Chlamydinae is an especially problematic group within the Pectinidae, perhaps partly because of the low sampling relative to the high number of species in the group. While Puslednik et al. [40] conclude that Chlamydinae—in contrast to Waller’s hypothesis (i.e., that Chlamydinae is paraphyletic, and have provided the ancestral stock for Palliolinae and Pectininae)—is in fact the crown group of the Pectinidae. However, their more recent study [31], doubled taxon sampling and used one more nuclear gene (28S), and subsequently came to the opposite conclusion, confirming Waller’s original hypothesis. Another, recent study by Smedley et al. [71] included yet another nuclear gene (18S), and—as they were studying the Pectinoidea—numerous outgroup species to Pectinidae. They revealed a similar topology to Alejandrino et al. [31], but with substantially lower support. This shows the importance of marker selection and appropriate taxon sampling on pectinid phylogenetics.

The phylogeny reconstructed from gene order data shows a similar, although not identical topology to the one reconstructed from sequence data. The difference between the two trees within the Pectininae can be explained with the likely incompleteness of the *Pe. albicans* mitogenome, missing a few tRNA genes. In accordance with our phylogenomic results, the gene order phylogeny fails to properly resolve Chlamydinae, which, again, can be attributed to low taxon sampling within this group.

The lack of sampling is even more prominent if we look at phylogenomic studies, as the number of published pectinid mitogenomes is currently low and only slowly growing. Although our study does not resolve this problem, it extends the list of published pectinid mitogenomes, shows the utility of mito-phylogenomics within the Pectinidae and expands our knowledge of the evolutionary history of mitochondria within this remarkable group of bivalves.

### Genome rearrangements

#### Gene collinearity

According to the ‘punctuation model’ [73], mitochondrial genomes are generally transcribed as a single polycistronic RNA from each strand, followed by the enzymatic removal of tRNAs, leading to gene-specific mRNAs [74]. In pectinids, major genes are not always separated by tRNAs (Fig. 6). It is possible that these cistrons are punctuated by secondary structures instead of tRNAs, or that they remain bicistrons. However, given that LCBs are the units of genome rearrangement, and most of them contain one major gene and some tRNA genes, it is probable that these are all separated during mRNA maturation. The one exception from this rule is LCB7, that contains the *nad6* and *cob* genes in every studied pectinid species but contains the *atp8* gene in addition to these in *Mim. varia* and Pectininae + Aequipectini, although in different position, and *atp8* together with *atp6* in *Pl. magellanicus* (Fig. 6, Additional file 3). Nevertheless, the variance in the composition of this block points towards it not being a single unit in rearrangements. The order of LCBs also shows a clear phylogenetic pattern. The divergence from this pattern in the case of the three outlier species, *Mim. varia*, *Miz. yessoensis* and *Pl. magellanicus* is most likely stemming from limited taxon sampling, i.e. they are relatively divergent from their closest relatives in our sampling, and their true close relatives would most likely show a similar gene order to theirs, as evidenced in other, better sampled groups in our study (e.g. *Argopecten*).

#### Common interval analysis

To the best of our knowledge, our study is the first attempt to reconstruct, at least partially, the ancestral pectinid mitogenome. The CREx method is widely used in other animal groups, for example insects, e.g. [75, 76]. While the ancestral gene order could be inferred for Insecta, shared by most groups, and some lineages showing some rearrangement, this is not the case for Mollusca. *Katharina tunicata* [17, 77] and *Solemya velum* [30] are hypothesized to carry a mitochondrial genome similar in organization to the ancestral Molluscan and Conchiferan mitogenomes, respectively. However, pectinid mitogenomes are very divergent from these two species, and from closely related groups with available mitogenomes, which severely limits the effectiveness of using these as outgroups, given the large chance for homoplasy. Our method tries to overcome this difficulty, with first reconstructing the putative gene order of internal nodes, including the common ancestor of the Pectinidae, followed by the inference of rearrangement events. Although some internal nodes were not consistent, we recovered the putative gene order of the common ancestor of the Chlamydinae, a monophyletic group in most phylogenetic analyses, including ours. While most previous studies focusing on mitochondrial genome rearrangements in the Pectinidae involved only a few species, we compare genome rearrangements among all available pectinid mitogenomes, with methods not previously used in this group. Confirming the assumption of Marín et al. [22], we have found that TDRL (Tandem Duplication/Random Loss) events are equally important as transpositions in pectinid mitogenome rearrangements.

## Conclusions

Scallop fisheries are commercially important and steadily growing worldwide [78, 79]. Considering that mitochondrial function is implicated in growth and environmental resilience [80, 81], it is important to understand the evolution of the unusual mitochondrial genomes of pectinids, as it would aid fisheries management in our changing climate.

In our study, we annotate and characterize the mitochondrial genome of *Mimachlamys varia*, we demonstrate that complete mitochondrial genomes are powerful resources in reconstructing pectinid phylogenies, and we also show the utility of several tools, not previously used in this group, to investigate mitochondrial genome rearrangements within the Pectinidae. We present annotations of the *atp8* gene in several pectinid species, where it was previously thought to be missing.

We show incongruities between phylogenies based on mitochondrial PCGs and rRNA genes, suggesting that locus sampling affects phylogenetic inference at the scale of the Pectinidae family. Both the lack of suitable outgroups—more closely related to the Pectinidae (e.g. Limidae, Entoliidae, Spondylidae and Propeamussiidae) than the currently available species—and the lack of sufficient taxon sampling, especially within Chlamydinae and Palliolinae, are limiting factors for current research. We therefore believe that sequencing and assembly of more mitogenomes, particularly from the aforementioned groups, would greatly improve the power of further research of both mito-phylogenomics and the evolution of mitogenome rearrangements in the Pectinidae.

## Methods

### Sequencing of the mitogenome of reference for the variegated scallop

One individual of *Mim. varia* (adult male, shell length: 46 mm, shell height: 40 mm) was collected in Angoulins, France, in order to sequence and annotate a reference transcriptome (nuclear and mitochondrial) for the species. RNA was purified from five tissues (digestive gland, mantle, gills, adductor muscle and gonads) using the Nucleospin RNA Set for Nucleozol kit (Macherey–Nagel). After quality control, extractions were pooled in equal amounts (4 μg of RNA per tissue type). The library was sequenced on Illumina HiSeq 2500 (2 × 300 bp). Full sample preparation and sequencing information is provided in Viricel et al. [50]; raw reads can be accessed on the NBCI Sequence Read Archive (Acc. SRP127478), and transcripts can be accessed at the Transcriptome Shotgun Assembly database (GGGO01000000).

Paired reads, minimally cleaned with Trimmomatic v. 0.36 ([82]; only adapters were removed as recommended in the NOVOPlasty manual), were used as input data for assembly with NOVOPlasty v. 4.2 [83] to assemble the mitochondrial genome. Quality-control before and after read filtering was done using FastQC v0.11.5 [84]. The size of the mitogenome being highly variable among pectinids (from 16,079 bp in *Argopecten ventricosus*, KT161261.1, to 32,115 bp in *Placopecten magellanicus*, NC_007234.1, [85]), we set a wide range for possible mitogenome sizes (15–35 kbp) as a parameter in NOVOPlasty. K-mer size was set to 39. We used a portion of the mitochondrial gene *cox1* as an initial seed for mitogenome assembly (we used the most common haplotype found along the coast of France, KU680872.1; [49]). When NOVOPlasty runs resulted in multiple contigs (mitogenome fragments), they were assembled in Geneious v2021.0.3 (https://www.geneious.com). Reads were mapped onto the assembly using bowtie2 v2.3.5.1 [86] and coverage statistics were generated with samtools v1.10 [87]. Once the reference mitogenome was assembled, we used NOVOPlasty to detect intra-individual heteroplasmy [88] using a minimum Minor Allele Frequency (MAF) of 0.1 and 0.01 in two separate runs. Finally, the MITOS2 web server (http://mitos2.bioinf.uni-leipzig.de/index.py) [89] was used to annotate the reference with default parameters, using the RefSeq 63 Metazoa database as reference and the protein-coding gene prediction method of Al Arab et al. [90]. The mitogenome was loaded into Geneious v2021.0.3. and checked manually for errors in annotations. ARWEN v1.2.3 [91], was used to look for additional tRNA genes and secondary structures of tRNAs were predicted using MITOS2 and the RNAfold web server, with default options. The Major Non-coding Region (MNR) was scanned for repeated regions using Tandem Repeats Finder v 4.09 (https://tandem.bu.edu/trf/trf.html) [92] and secondary structures were predicted using the mfold Web Server (http://www.unafold.org/) [93]. The circular mitogenome was visualised using the CGView Web Server (http://cgview.ca/) [94], with annotations imported from Geneious.

### Assembly and annotation of pectinid mitogenomes from GenBank

All currently available pectinid mitogenomes (as of January 2021) were downloaded from GenBank (Table 4) along with *Ostrea lurida* (Ostreidae) as an outgroup. We chose an oyster species as outgroup as there are no published mitogenomes of taxa more closely related to Pectinidae. Using only one outgroup made interpreting comparisons of mitogenome rearrangements substantially less difficult. The largest mitogenome from each species was reannotated using the MITOS2 web server with the same settings as for the *Mim. varia* mitogenome. New annotations of pectinid mitogenomes were reviewed manually, in the same way as it was described for *Mim. varia.* If there were multiple annotations for a gene, they were filtered manually based on length and sequence similarity to other species. Additionally, tRNA genes were compared with previously published annotations. Next, this reference sequence of each species was used to annotate other sequences of the same species using the “Live Annotate & Predict” feature in Geneious.

### Mito-phylogenomics

Phylogenetic analysis was conducted on the 13 protein coding genes (PCGs) and two Ribosomal RNA (rRNA) genes of all available pectinid species in GenBank, *Mim. varia* and *O. lurida* as an outgroup. This approach provides the most comprehensive dataset without the ambiguities arising from tRNA annotations, non-coding regions and genome rearrangements. Every gene was extracted and aligned separately in Geneious v2021.0.3. In the case of the *atp8* gene, where more copies were identified in some species, a maximum likelihood (ML) tree was built with the RAxML [95] plugin of Geneious using default parameters, and the least divergent copy was selected for further analysis. The *atp8* gene of two accessions of *Argopecten irradians* (EU023915; NC_009687) were not included in the phylogenetic analyses given their unusual structure, and divergent sequences. The 13 PCG sequences were aligned separately based on translated amino-acids with MAFFT [96] with the FFT-NS-2 algorithm as implemented in TranslatorX [97]. As the initial alignments contained indels and poorly aligned regions, these regions were masked with Gblocks [98] as implemented in TranslatorX, with default settings (i.e., allowing smaller final blocks, gap positions within the final blocks and less strict flanking positions). The two rRNA genes were aligned with MAFFT in Geneious using the FFT-NS-2 algorithm. Regions of uncertainty in the alignment were then masked with Gblocks using the same settings as for the PCGs. Individual gene alignments were then concatenated in Geneious resulting in eight datasets used for phylogenetic analyses: (A) all PCG and rRNA genes treated with Gblocks; (B) all PCG amino acid sequences and rRNA genes treated with Gblocks; (C) all PCGs; (D) all PCGs treated with Gblocks; (E) all PCG amino acid sequences; (F) all PCG amino acid sequences treated with Gblocks; (G) rRNA genes; (H) rRNA genes treated with Gblocks. These datasets were prepared in order to investigate the effect of rRNA and protein-coding genes as well as the presence/absence of ambiguously aligned regions on the topology and branch support of the phylogeny.

Maximum likelihood phylogenetic reconstruction was achieved using IQtree v2.0.6 [99] (except for the B dataset, where IQtree v1.6.12 was used), with automatic model selection and using the best partition scheme in ModelFinder (option MFP + MERGE) [100], 10,000 Ultrafast Bootstrap replicates and 10,000 replicates of the SH-like approximate likelihood ratio test (SH-aLRT) [101] on all eight datasets. Partitioning and substitution model information for every run can be found in Additional file 6.

Bayesian analyses were done using MrBayes v3.2.7 [102]. For each dataset, two runs were performed over 1.5 million generations, with four chains each (three heated and one cold). Trees were sampled every 500 generations, and the first 25% were discarded as burn-in. Finally, a majority consensus tree was constructed to estimate posterior probabilities of branches. Convergence of each run was evaluated through effective sample size (ESS) values and trace plots in the software Tracer v.1.6 [103]. Partitioning and prior model parameter settings can be found in Additional file 7.

### Genome rearrangements

Three approaches were used to infer mitogenome rearrangements in the Pectinidae: (1) gene collinearity analysis, which detects mosaic patterns of homology among a set of genomes, based on whole-genome sequence alignment; (2) common interval analysis, which determines pairwise rearrangement events between genomes, based on gene order data, considering several rearrangement types, but requiring identical gene content; and (3) maximum likelihood analysis of gene orders, which reconstructs phylogenies based solely on gene order data, without requiring an identical set of genes between genomes.

#### Gene collinearity

Gene collinearity among pectinid mitogenomes (one individual/species, 13 individuals in total) was explored using the progressiveMauve algorithm and default parameters [automatically calculating seed weight and the minimum LCB score, computing Locally Collinear Blocks (LCBs) and using full alignments] in the MAUVE Geneious plugin [104].

#### Common interval analysis

Two joint softwares—both relying on the same algorithm—CREx [105] and TreeREx [106] were implemented for common interval analyses. Both software consider the following types of rearrangement events: transpositions, inversions, inverse transpositions and tandem-duplication/random loss (TDRL) involving the duplication of a gene block followed by the random loss of one copy of a gene in either of the two blocks. Both tools are only able to handle identical gene sets among species. To conform to this condition, only major genes (PCGs and rRNA genes) and the MNR were included in this analysis, as tRNA gene content is variable among pectinid mitogenomes.

The MrBayes tree based on the most supported dataset (B), with only eight pectinids and one outgroup species representing unique gene orders, along with the gene order dataset described above, was used in the first step, using the TreeREx [106] software. TreeREx uses the phylogenetic information in the tree to ascertain rearrangement events among nodes of the tree, and to infer putative gene orders of common ancestors at internal nodes. TreeREx was used with default settings: strong consistency method applied (-s); weak consistency method applied (-w); parsimonious weak consistency method applied (-W); and the maximum number of inversions (-m) set to 2. Finally, the input dataset in the previous analysis, complemented with the putative gene orders inferred at internal nodes was put into CREx again, to visualize each rearrangement event between each pair of neighboring nodes.

#### Maximum likelihood analysis of gene orders

The MLGO (Maximum Likelihood for Gene-Order Analysis) [107] web server can be used to directly infer a phylogeny based on gene order data. The algorithm handles insertions, deletions and duplications along with transpositions. Unlike CREx/TreeREx, it is not limited to a common set of genes across taxa. Complete gene order data from 13 pectinids (one individual per species) and one outgroup (*O. lurida*) were used to infer a phylogeny of the Pectinidae. Branch support was computed with a bootstrap analysis of 1000 replicates.

## Supplementary Information

**Additional file 1.** Putative secondary structures of mitochondrial tRNAs in *Mimachlamys varia*. Amino acids are represented by their one-letter code. (*: structure predicted by RNAfold, all other structures were predicted by MITOS2)

**Additional file 2. **Putative secondary structure of the Major Non-coding Region of *Mimachlamys varia*. There is a 100 basepair overlap kept between parts. 1: 5484–6439; 2: 6339–7805; 3: 7705–9689.

**Additional file 3. **Annotation tables for species involved in this study.

**Additional file 4. **Common interval analysis results presented on a Bayesian tree, where each tip represents a unique gene order. Internal nodes are named as An, where n is a number between 0 and 7, and represent ancestral gene orders. C and P indicate consistency and parsimony values, respectively. The colors code consistent (green; highest level of certainty), k-consistent (yellow; intermediate level of certainty) and inconsistent (red; low level of certainty) nodes. Mutation types are indicated on corresponding branches, T: transposition, TDRL: tandem duplication and random loss. Arrows point to diagrams showing the two gene orders, and the mutation steps leading from the more ancestral to the younger node. Frames connect gene blocks affected by a rearrangement event, and parts of the mitogenome that is affected by a rearrangement event.

**Additional file 5. **Tandem repeats detected in pectinid mitogenomes.

**Additional file 6.** Partitioning and substitution model information for all eight datasets used in the Maximum Likelihood analyses.

**Additional file 7.** Partitioning schemes and prior model parameter settings for all eight datasets used in the Bayesian analyses.

## Data Availability

Raw *Mimachlamys varia* RNAseq reads are deposited in the NBCI Sequence Read Archive (Acc. SRP127478), and transcripts can be accessed at the Transcriptome Shotgun Assembly database (GGGO01000000). The assembled mitochondrial genome is uploaded to GenBank (MZ520326), and the GenBank accession numbers of all mitogenomes used in this study can be found in Table 4. The sequence alignments used in this article are publicly available on GitHub (https://github.com/ericpante/Malkocs_etal_2022).
