# Supplementary material for: Complex mitogenomic rearrangements within the Pectinidae (Mollusca: Bivalvia)
Source: BMC Ecol Evol. 2022 Mar 10;22:29. doi: 10.1186/s12862-022-01976-0 (PMC8915466; doi:10.1186/s12862-022-01976-0)
Supplement: Supplementary file 1 — Additional file 1. Putative secondary structures of mitochondrial tRNAs in Mimachlamys varia. Amino acids are represented by their one-letter code. (*: structure predicted by RNAfold, all other structures were predicted by MITOS2) [file 12862_2022_1976_MOESM1_ESM.pdf]

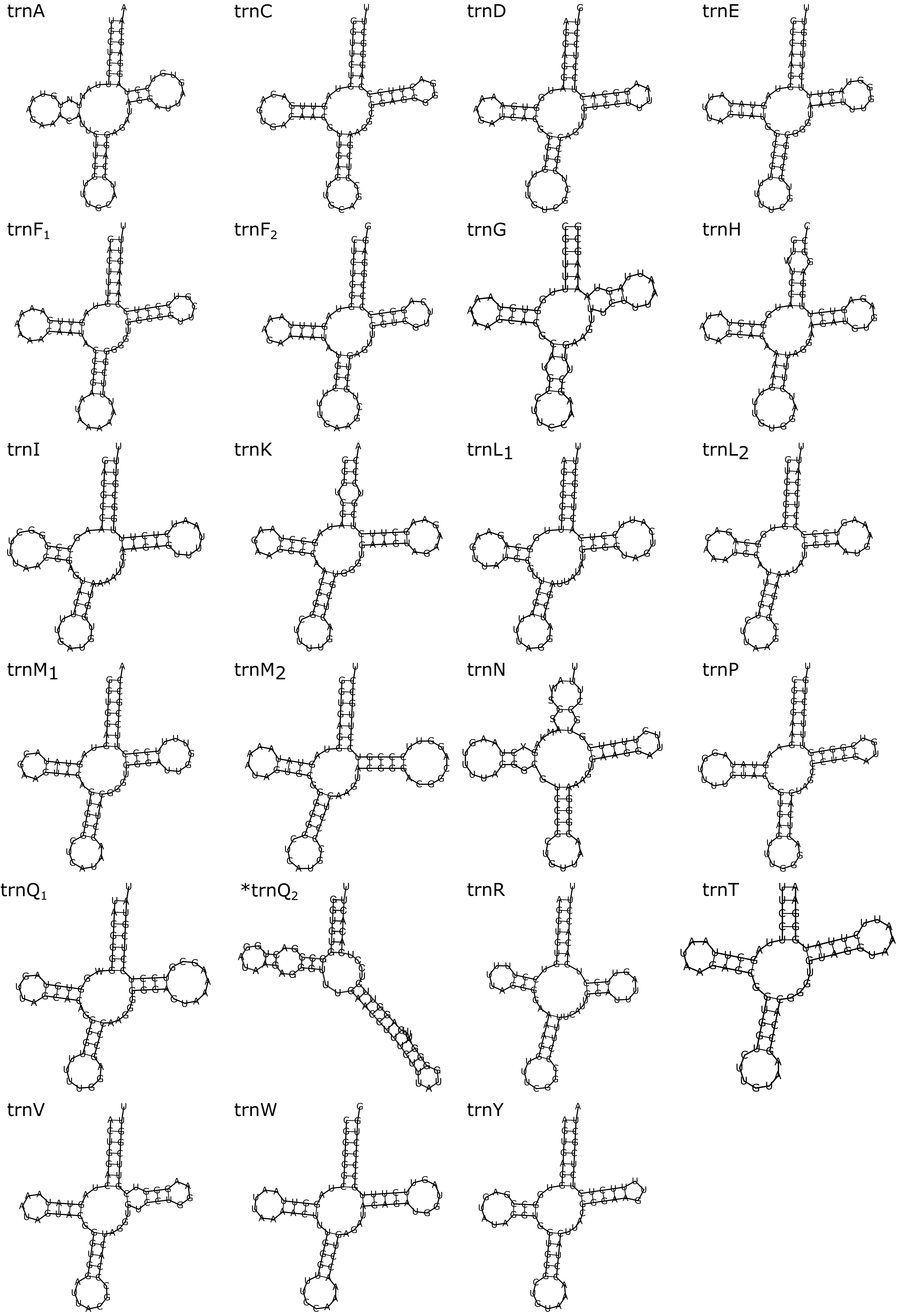
trnA
trnC
trnD
trnE
trnF1
trnF2
trnG
trnH
trnI
trnK
trnL1
trnL2
trnM1
trnM2
trnN
trnP
trnQ1
*trnQ2
trnR
trnT
trnV
trnW
trnY
