# Supplementary figures and images for: Complex mitogenomic rearrangements within the Pectinidae (Mollusca: Bivalvia)
Source: BMC Ecol Evol. 2022 Mar 10;22:29. doi: 10.1186/s12862-022-01976-0 (PMC8915466; doi:10.1186/s12862-022-01976-0)

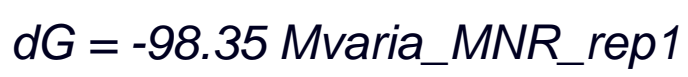

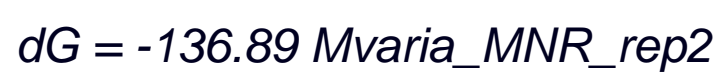

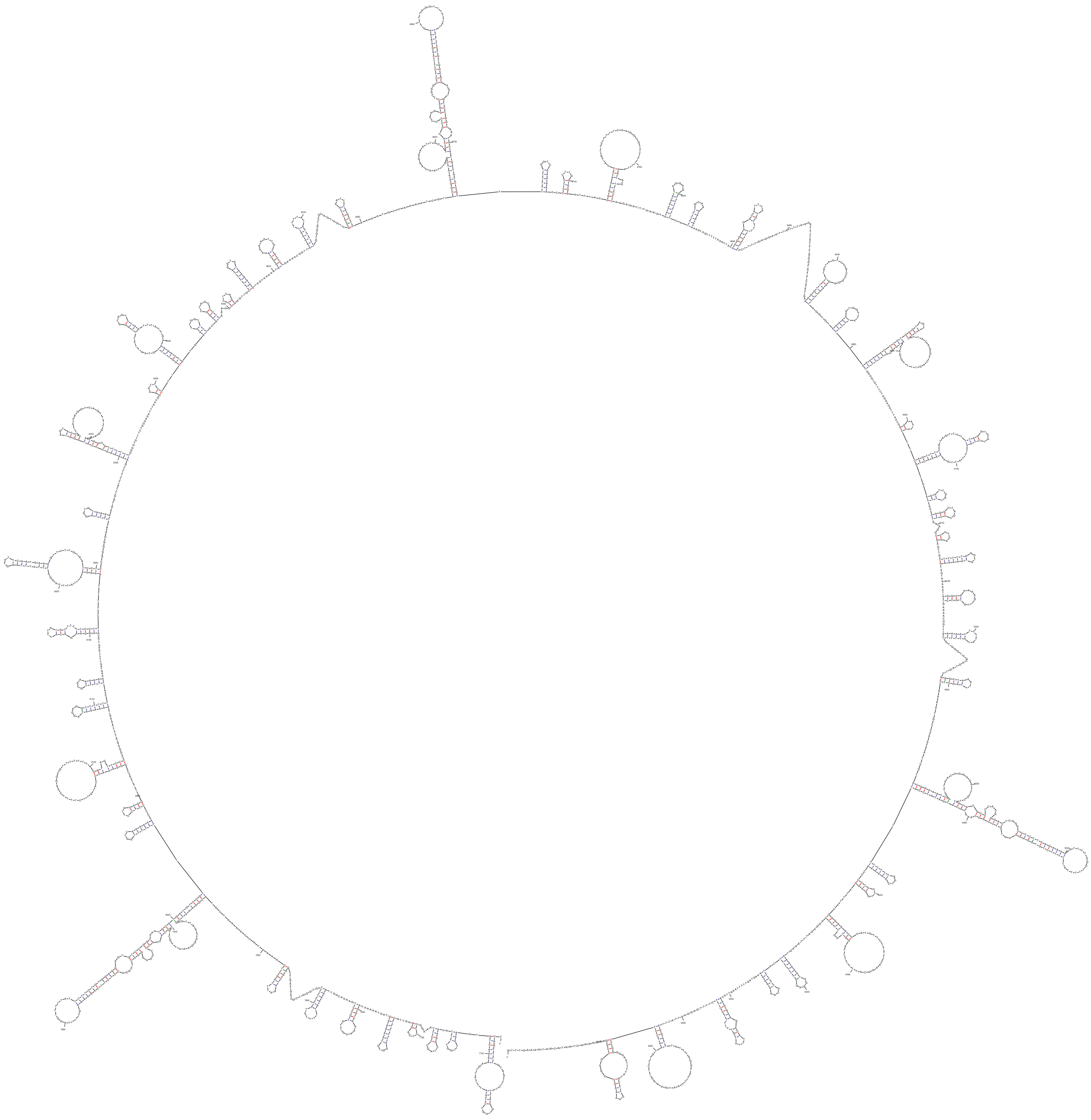

*dG = -188.23 M varia\_MNR\_rep3*

Supplement: Supplementary file 2 — Additional file 2. Putative secondary structure of the Major Non-coding Region of Mimachlamys varia. There is a 100 basepair overlap kept between parts. 1: 5484–6439; 2: 6339–7805; 3: 7705–9689. [file 12862_2022_1976_MOESM2_ESM.pdf]

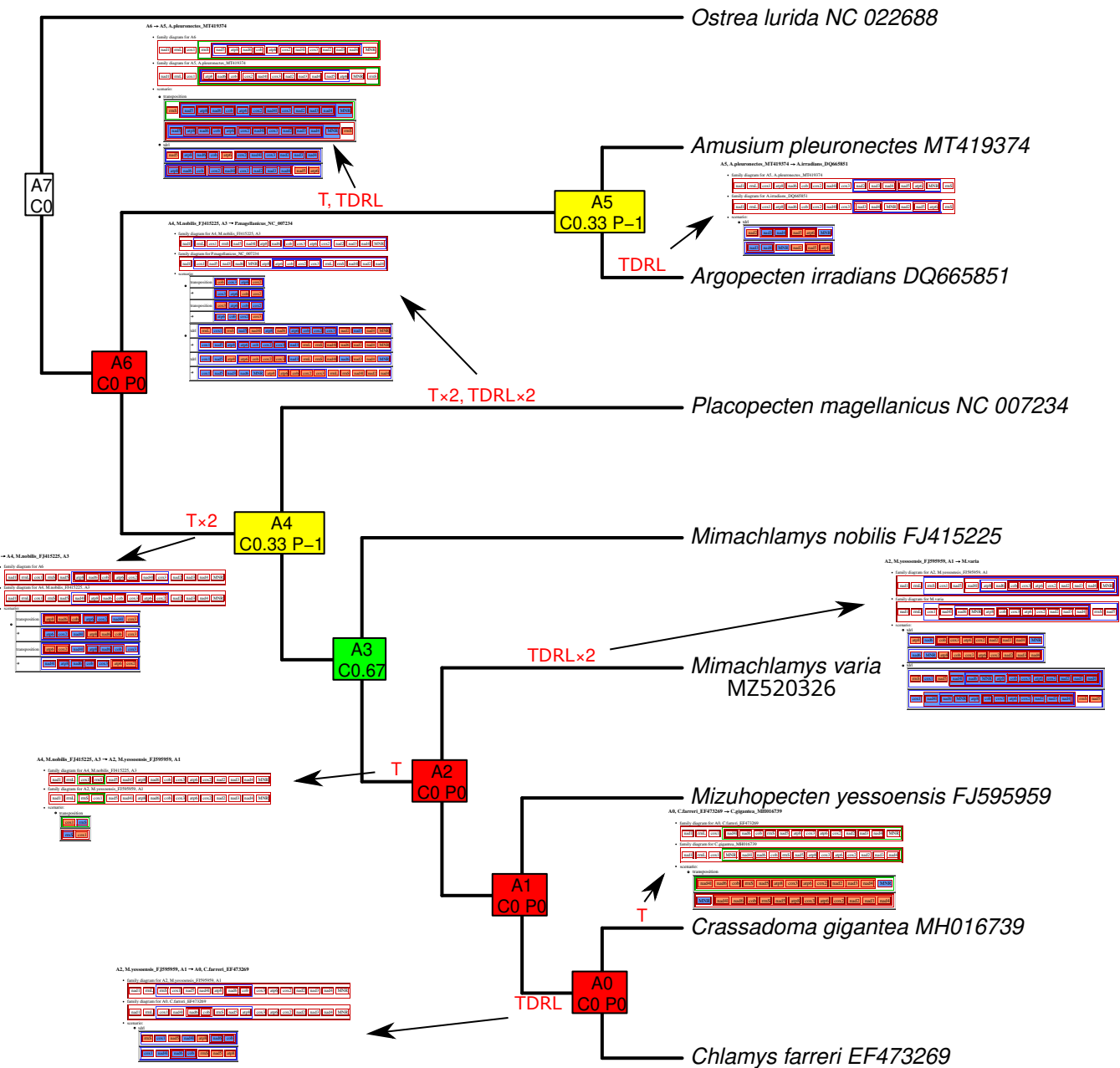

Supplement: Supplementary file 4 — Additional file 4. Common interval analysis results presented on a Bayesian tree, where each tip represents a unique gene order. Internal nodes are named as An, where n is a number between 0 and 7, and represent ancestral gene orders. C and P indicate consistency and parsimony values, respectively. The colors code consistent (green; highest level of certainty), k-consistent (yellow; intermediate level of certainty) and inconsistent (red; low level of certainty) nodes. Mutation types are indicated on corresponding branches, T: transposition, TDRL: tandem duplication and random loss. Arrows point to diagrams showing the two gene orders, and the mutation steps leading from the more ancestral to the younger node. Frames connect gene blocks affected by a rearrangement event, and parts of the mitogenome that is affected by a rearrangement event. [file 12862_2022_1976_MOESM4_ESM.pdf]
