# Supplementary material for: Complex mitogenomic rearrangements within the Pectinidae (Mollusca: Bivalvia)
Source: BMC Ecol Evol. 2022 Mar 10;22:29. doi: 10.1186/s12862-022-01976-0 (PMC8915466; doi:10.1186/s12862-022-01976-0)
Supplement: Supplementary file 3 — Additional file 3. Annotation tables for species involved in this study. [file 12862_2022_1976_MOESM3_ESM.docx]

Annotation table for *Placopecten magellanicus*.

| Species | Start | Stop | Gene | Score | Strand |
| --- | --- | --- | --- | --- | --- |
| *Pl. magellanicus* | 2 | 68 | trnG(tcc) | 5.10E-06 | + |
| *Pl. magellanicus* | 63 | 231 | atp8_2 | 0 | + |
| *Pl. magellanicus* | 1853 | 1923 | trnM_0(cat) | 2.80E-08 | + |
| *Pl. magellanicus* | 2032 | 2102 | trnM_3(cat) | 9.90E-07 | + |
| *Pl. magellanicus* | 2105 | 2172 | trnN_0(gtt) | 3.00E-05 | + |
| *Pl. magellanicus* | 3108 | 3175 | trnE(ttc) | 3.90E-05 | + |
| *Pl. magellanicus* | 4109 | 4176 | trnD(gtc) | 1.50E-02 | + |
| *Pl. magellanicus* | 4675 | 4743 | trnK(uuu) | 0 | + |
| *Pl. magellanicus* | 5716 | 5782 | trnS(nga) | 0 | + |
| *Pl. magellanicus* | 6138 | 6206 | trnR(ucg) | 0 | + |
| *Pl. magellanicus* | 6211 | 7610 | rrnL | 0 | + |
| *Pl. magellanicus* | 7691 | 8667 | rrnS | 0 | + |
| *Pl. magellanicus* | 9165 | 9233 | trnS(nga) | 0 | + |
| *Pl. magellanicus* | 9651 | 9948 | nad4l | 15.8 | + |
| *Pl. magellanicus* | 10004 | 10916 | nad2 | 48.9 | + |
| *Pl. magellanicus* | 11008 | 12292 | nad4 | 106.3 | + |
| *Pl. magellanicus* | 12294 | 12361 | trnH(gtg) | 3.20E-04 | + |
| *Pl. magellanicus* | 12369 | 12436 | trnW(cca) | 1.90E-06 | + |
| *Pl. magellanicus* | 12437 | 12503 | trnY(gta) | 1.20E-12 | + |
| *Pl. magellanicus* | 12512 | 12579 | trnT(tgt) | 1.10E-04 | + |
| *Pl. magellanicus* | 12588 | 12655 | trnP(tgg) | 2.70E-05 | + |
| *Pl. magellanicus* | 12656 | 12727 | trnI(gat) | 1.00E-04 | + |
| *Pl. magellanicus* | 12732 | 12802 | trnM_9(cat) | 2.90E-04 | + |
| *Pl. magellanicus* | 12815 | 12883 | trnC(gca) | 3.00E-06 | + |
| *Pl. magellanicus* | 12888 | 12954 | trnL1(tag) | 3.20E-07 | + |
| *Pl. magellanicus* | 12958 | 13027 | trnM(uau) | 0 | + |
| *Pl. magellanicus* | 13036 | 13106 | trnV(uac) | 0 | + |
| *Pl. magellanicus* | 13107 | 14055 | nad1 | 101.4 | + |
| *Pl. magellanicus* | 14251 | 15793 | cox1 | 207.8 | + |
| *Pl. magellanicus* | 16017 | 17712 | nad5 | 158.4 | + |
| *Pl. magellanicus* | 17732 | 18080 | nad3 | 27 | + |
| *Pl. magellanicus* | 18083 | 18150 | trnS(uga) | 0 | + |
| *Pl. magellanicus* | 18150 | 18218 | trnM(uau) | 0 | + |
| *Pl. magellanicus* | 18283 | 18748 | nad6 | 14.2 | + |
| *Pl. magellanicus* | 19143 | 19209 | trnM_5(tat) | 2.10E-06 | + |
| *Pl. magellanicus* | 20578 | 20644 | trnM_6(tat) | 2.10E-06 | + |
| *Pl. magellanicus* | 22321 | 22389 | trnF(gaa) | 0 | + |
| *Pl. magellanicus* | 22541 | 22611 | trnM_1(tat) | 3.50E-08 | + |
| *Pl. magellanicus* | 22768 | 22836 | trnF(gaa) | 0 | + |
| *Pl. magellanicus* | 23432 | 23498 | trnM_8(tat) | 2.60E-05 | + |
| *Pl. magellanicus* | 23483 | 23660 | atp8_1 | 1.4 | + |
| *Pl. magellanicus* | 23770 | 24451 | atp6 | 40.3 | + |
| *Pl. magellanicus* | 25050 | 25114 | trnL2(taa) | 1.80E-05 | + |
| *Pl. magellanicus* | 25215 | 26304 | cob | 136.1 | + |
| *Pl. magellanicus* | 26403 | 27088 | cox2 | 66.3 | + |
| *Pl. magellanicus* | 28107 | 28178 | trnQ_0(ttg) | 2.60E-07 | + |
| *Pl. magellanicus* | 28190 | 28257 | trnE(uuc) | 0 | + |
| *Pl. magellanicus* | 28284 | 28352 | trnK(ttt) | 1.60E-06 | + |
| *Pl. magellanicus* | 28358 | 28422 | trnF(gaa) | 4.00E-06 | + |
| *Pl. magellanicus* | 28422 | 28488 | trnA(tgc) | 5.70E-09 | + |
| *Pl. magellanicus* | 28574 | 29361 | cox3 | 90.2 | + |
| *Pl. magellanicus* | 29639 | 29707 | trnF(gaa) | 0 | + |
| *Pl. magellanicus* | 29859 | 29929 | trnM_2(tat) | 3.50E-08 | + |
| *Pl. magellanicus* | 30653 | 30719 | trnM_7(tat) | 2.10E-06 | + |
| *Pl. magellanicus* | 31354 | 31424 | trnM_4(cat) | 9.90E-07 | + |
| *Pl. magellanicus* | 31427 | 31494 | trnN_1(gtt) | 3.00E-05 | + |

Annotation table for *Chlamys farreri*.

| Species | Start | Stop | Gene | Score | Strand |
| --- | --- | --- | --- | --- | --- |
| *Ch. farreri* | 0 | 1536 | cox1 | 215.2 | + |
| *Ch. farreri* | 1825 | 2101 | nad4l | 17.1 | + |
| *Ch. farreri* | 2136 | 2634 | nad6 | 14.4 | + |
| *Ch. farreri* | 2640 | 2704 | trnL2(taa) | 7.20E-08 | + |
| *Ch. farreri* | 2971 | 4057 | cob | 114.7 | + |
| *Ch. farreri* | 4075 | 4145 | trnC(gca) | 3.00E-08 | + |
| *Ch. farreri* | 4162 | 4229 | trnA(tgc) | 6.40E-08 | + |
| *Ch. farreri* | 4262 | 5220 | rrnS | 0 | + |
| *Ch. farreri* | 5385 | 7053 | nad5 | 165.3 | + |
| *Ch. farreri* | 7070 | 7217 | atp8 | 2.1 | + |
| *Ch. farreri* | 7263 | 7329 | trnD(gtc) | 4.80E-04 | + |
| *Ch. farreri* | 7332 | 8184 | cox3 | 82.9 | + |
| *Ch. farreri* | 8224 | 8294 | trnK(ttt) | 8.00E-09 | + |
| *Ch. farreri* | 8300 | 8364 | trnF(gaa) | 4.50E-07 | + |
| *Ch. farreri* | 8371 | 8438 | trnS2(tga) | 7.00E-03 | + |
| *Ch. farreri* | 8441 | 8512 | trnQ(ttg) | 9.40E-09 | + |
| *Ch. farreri* | 8518 | 8585 | trnE(ttc) | 6.30E-04 | + |
| *Ch. farreri* | 8711 | 9380 | atp6 | 37.7 | + |
| *Ch. farreri* | 9487 | 10149 | cox2 | 60.6 | + |
| *Ch. farreri* | 10525 | 11494 | nad2 | 52.9 | + |
| *Ch. farreri* | 11512 | 11580 | trnT(tgt) | 2.90E-05 | + |
| *Ch. farreri* | 11589 | 11657 | trnP(tgg) | 1.90E-05 | + |
| *Ch. farreri* | 11663 | 11733 | trnI(gat) | 1.20E-07 | + |
| *Ch. farreri* | 11734 | 11801 | trnL1(tag) | 9.40E-07 | + |
| *Ch. farreri* | 11803 | 11872 | trnM(cau) | 0 | + |
| *Ch. farreri* | 11815 | 12166 | nad3 | 23 | + |
| *Ch. farreri* | 12233 | 12301 | trnS(ucu) | 0 | + |
| *Ch. farreri* | 12344 | 13589 | nad4 | 116.2 | + |
| *Ch. farreri* | 13590 | 13654 | trnH(gtg) | 1.80E-03 | + |
| *Ch. farreri* | 13667 | 13736 | trnW(cca) | 1.80E-06 | + |
| *Ch. farreri* | 13737 | 13804 | trnY(gta) | 2.80E-11 | + |
| *Ch. farreri* | 13806 | 13869 | trnG(tcc) | 9.70E-07 | + |
| *Ch. farreri* | 13894 | 13960 | trnV(tac) | 5.30E-02 | + |
| *Ch. farreri* | 17919 | 17985 | trnN(gtt) | 5.40E-04 | + |
| *Ch. farreri* | 17997 | 18936 | nad1 | 104.4 | + |
| *Ch. farreri* | 18938 | 19003 | trnR(tcg) | 1.10E-02 | + |
| *Ch. farreri* | 19034 | 20489 | rrnL | 0 | + |
| *Ch. farreri* | 20695 | 20768 | trnM(cat) | 1.00E-05 | + |

Annotation table for *Mizuhopecten yessoensis*.

| Species | Start | Stop | Gene | Score | Strand |
| --- | --- | --- | --- | --- | --- |
| *Miz. yessoensis* | 0 | 66 | trnN(gtt) | 8.80E-05 | + |
| *Miz. yessoensis* | 78 | 1017 | nad1 | 105.2 | + |
| *Miz. yessoensis* | 1021 | 1086 | trnR(tcg) | 1.20E-03 | + |
| *Miz. yessoensis* | 1092 | 2577 | rrnL | 0 | + |
| *Miz. yessoensis* | 2599 | 2671 | trnM_0(cat) | 3.00E-06 | + |
| *Miz. yessoensis* | 2890 | 3851 | rrnS | 0 | + |
| *Miz. yessoensis* | 4629 | 6174 | cox1 | 214.2 | + |
| *Miz. yessoensis* | 6228 | 6295 | trnS(aga) | 0 | + |
| *Miz. yessoensis* | 7275 | 7345 | trnC(gca) | 2.40E-07 | + |
| *Miz. yessoensis* | 7588 | 9202 | nad5 | 168.1 | + |
| *Miz. yessoensis* | 9238 | 9385 | atp8_2 | 0 | + |
| *Miz. yessoensis* | 9447 | 9726 | nad4l | 1.72E+01 | + |
| *Miz. yessoensis* | 9796 | 9943 | atp8_1 | 2.70E+00 | + |
| *Miz. yessoensis* | 9956 | 10022 | trnD(gtc) | 1.20E-03 | + |
| *Miz. yessoensis* | 10062 | 10560 | nad6 | 1.52E+01 | + |
| *Miz. yessoensis* | 10567 | 10631 | trnL2(taa) | 9.30E-07 | + |
| *Miz. yessoensis* | 10838 | 11972 | cob | 116.2 | + |
| *Miz. yessoensis* | 12547 | 12616 | trnD(guc) | 0 | + |
| *Miz. yessoensis* | 12616 | 13403 | cox3 | 88 | + |
| *Miz. yessoensis* | 13489 | 13559 | trnK(ttt) | 2.30E-07 | + |
| *Miz. yessoensis* | 13567 | 13632 | trnF(gaa) | 2.40E-06 | + |
| *Miz. yessoensis* | 13636 | 13703 | trnS2(tga) | 8.50E-02 | + |
| *Miz. yessoensis* | 13706 | 13776 | trnQ(ttg) | 7.10E-09 | + |
| *Miz. yessoensis* | 13781 | 13847 | trnE(ttc) | 3.00E-05 | + |
| *Miz. yessoensis* | 13862 | 14597 | atp6 | 38.5 | + |
| *Miz. yessoensis* | 14766 | 15454 | cox2 | 5.48E+01 | + |
| *Miz. yessoensis* | 15748 | 16711 | nad2 | 55.2 | + |
| *Miz. yessoensis* | 16729 | 16797 | trnT(tgt) | 3.30E-07 | + |
| *Miz. yessoensis* | 16807 | 16875 | trnP(tgg) | 1.40E-06 | + |
| *Miz. yessoensis* | 16877 | 16947 | trnI(gat) | 8.80E-09 | + |
| *Miz. yessoensis* | 16949 | 17015 | trnL1(tag) | 2.20E-05 | + |
| *Miz. yessoensis* | 17019 | 17085 | trnM_1(cat) | 3.00E-06 | + |
| *Miz. yessoensis* | 17095 | 17449 | nad3 | 2.89E+01 | + |
| *Miz. yessoensis* | 17453 | 17521 | trnS(ucu) | 0 | + |
| *Miz. yessoensis* | 17568 | 18813 | nad4 | 116.3 | + |
| *Miz. yessoensis* | 18819 | 18887 | trnV(uac) | 0 | + |

Annotation table for *Crassadoma gigantea*.

| Species | Start | Stop | Gene | Score | Strand |
| --- | --- | --- | --- | --- | --- |
| *Cr. gigantea* | 226 | 292 | trnN(gtt) | 1.50E-05 | + |
| *Cr. gigantea* | 309 | 1248 | nad1 | 104.1 | + |
| *Cr. gigantea* | 1252 | 1318 | trnR(tcg) | 5.10E-04 | + |
| *Cr. gigantea* | 1325 | 2804 | rrnL | 0 | + |
| *Cr. gigantea* | 2822 | 2890 | trnM_1(cat) | 6.90E-06 | + |
| *Cr. gigantea* | 3125 | 4682 | cox1 | 198.8 | + |
| *Cr. gigantea* | 5903 | 6179 | nad4l | 1.76E+01 | + |
| *Cr. gigantea* | 6232 | 6733 | nad6 | 1.52E+01 | + |
| *Cr. gigantea* | 6735 | 6799 | trnL(uaa) | 0 | + |
| *Cr. gigantea* | 6748 | 7883 | cob | 138.6 | + |
| *Cr. gigantea* | 8180 | 8250 | trnC(gca) | 1.10E-07 | + |
| *Cr. gigantea* | 8259 | 8326 | trnA(tgc) | 9.50E-09 | + |
| *Cr. gigantea* | 8343 | 9307 | rrnS | 0 | + |
| *Cr. gigantea* | 9401 | 11123 | nad5 | 164.1 | + |
| *Cr. gigantea* | 11144 | 11303 | atp8 | 0 | + |
| *Cr. gigantea* | 11319 | 11385 | trnD(gtc) | 2.80E-03 | + |
| *Cr. gigantea* | 11461 | 12250 | cox3 | 81.1 | + |
| *Cr. gigantea* | 12316 | 12386 | trnK(ttt) | 1.70E-07 | + |
| *Cr. gigantea* | 12394 | 12458 | trnF(gaa) | 1.10E-07 | + |
| *Cr. gigantea* | 12463 | 12529 | trnS2(tga) | 6.70E-04 | + |
| *Cr. gigantea* | 12532 | 12602 | trnQ(ttg) | 8.00E-07 | + |
| *Cr. gigantea* | 12611 | 12677 | trnE(ttc) | 6.00E-06 | + |
| *Cr. gigantea* | 12803 | 13469 | atp6 | 37 | + |
| *Cr. gigantea* | 13516 | 14192 | cox2 | 64.4 | + |
| *Cr. gigantea* | 14765 | 15776 | nad2 | 54.8 | + |
| *Cr. gigantea* | 15785 | 15847 | trnT(tgt) | 3.80E-05 | + |
| *Cr. gigantea* | 15859 | 15924 | trnP(tgg) | 8.20E-06 | + |
| *Cr. gigantea* | 15928 | 15998 | trnI(gat) | 6.50E-09 | + |
| *Cr. gigantea* | 15999 | 16066 | trnL1(tag) | 3.80E-07 | + |
| *Cr. gigantea* | 16068 | 16133 | trnM_0(cat) | 4.60E-07 | + |
| *Cr. gigantea* | 16141 | 16495 | nad3 | 2.91E+01 | + |
| *Cr. gigantea* | 16503 | 16573 | trnS(ucu) | 0 | + |
| *Cr. gigantea* | 16616 | 17861 | nad4 | 117.7 | + |
| *Cr. gigantea* | 17863 | 17927 | trnH(gtg) | 2.20E-04 | + |
| *Cr. gigantea* | 17934 | 18003 | trnW(cca) | 1.20E-06 | + |
| *Cr. gigantea* | 18004 | 18071 | trnY(gta) | 7.10E-12 | + |
| *Cr. gigantea* | 18071 | 18137 | trnG(tcc) | 9.20E-07 | + |
| *Cr. gigantea* | 18167 | 18233 | trnV(uac) | 0 | + |

Annotation table for *Mimachlamys nobilis*.

| Species | Start | Stop | Gene | Score | Strand |
| --- | --- | --- | --- | --- | --- |
| *Mim. nobilis* | 259 | 325 | trnN(gtt) | 1.70E-04 | + |
| *Mim. nobilis* | 333 | 401 | trnT(ugu) | 0.00E+00 | + |
| *Mim. nobilis* | 451 | 514 | trnG(tcc) | 1.50E-05 | + |
| *Mim. nobilis* | 527 | 596 | trnV(uac) | 0.00E+00 | + |
| *Mim. nobilis* | 610 | 1549 | nad1 | 101.7 | + |
| *Mim. nobilis* | 1553 | 1619 | trnR(tcg) | 5.20E-04 | + |
| *Mim. nobilis* | 1644 | 3052 | rrnL | 0 | + |
| *Mim. nobilis* | 3299 | 4856 | cox1 | 220.1 | + |
| *Mim. nobilis* | 4918 | 4984 | trnC(gca) | 1.40E-06 | + |
| *Mim. nobilis* | 4999 | 5066 | trnA(tgc) | 3.40E-08 | + |
| *Mim. nobilis* | 5084 | 6029 | rrnS | 0 | + |
| *Mim. nobilis* | 6194 | 7829 | nad5 | 161 | + |
| *Mim. nobilis* | 7904 | 8180 | nad4l | 16.8 | + |
| *Mim. nobilis* | 8196 | 8349 | atp8 | 5.1 | + |
| *Mim. nobilis* | 8399 | 8472 | trnM_1(cat) | 3.80E-06 | + |
| *Mim. nobilis* | 8513 | 9008 | nad6 | 18.1 | + |
| *Mim. nobilis* | 9011 | 9076 | trnL2(taa) | 4.30E-06 | + |
| *Mim. nobilis* | 9130 | 10207 | cob | 146.6 | + |
| *Mim. nobilis* | 10526 | 10595 | trnD(gtc) | 6.80E-06 | + |
| *Mim. nobilis* | 10645 | 11461 | cox3 | 79.1 | + |
| *Mim. nobilis* | 11502 | 11571 | trnK(ttt) | 1.90E-07 | + |
| *Mim. nobilis* | 11575 | 11638 | trnF(gaa) | 2.90E-10 | + |
| *Mim. nobilis* | 11641 | 11713 | trnS(uga) | 0.00E+00 | + |
| *Mim. nobilis* | 11712 | 11783 | trnQ(ttg) | 7.30E-07 | + |
| *Mim. nobilis* | 11790 | 11856 | trnE(ttc) | 8.90E-05 | + |
| *Mim. nobilis* | 11859 | 12552 | atp6 | 43.1 | + |
| *Mim. nobilis* | 12859 | 13465 | cox2 | 55.3 | + |
| *Mim. nobilis* | 13550 | 14516 | nad2 | 50.3 | + |
| *Mim. nobilis* | 14528 | 14837 | nad3 | 19.8 | + |
| *Mim. nobilis* | 14895 | 14964 | trnS1(tct) | 0.00E+00 | + |
| *Mim. nobilis* | 14970 | 16251 | nad4 | 111.1 | + |
| *Mim. nobilis* | 16252 | 16316 | trnH(gtg) | 1.40E-04 | + |
| *Mim. nobilis* | 16323 | 16390 | trnW(cca) | 4.40E-07 | + |
| *Mim. nobilis* | 16391 | 16457 | trnY(gta) | 6.40E-14 | + |
| *Mim. nobilis* | 16475 | 16546 | trnT(tgt) | 2.50E-10 | + |
| *Mim. nobilis* | 16549 | 16616 | trnP(tgg) | 2.80E-06 | + |
| *Mim. nobilis* | 16619 | 16689 | trnI(gat) | 1.30E-07 | + |
| *Mim. nobilis* | 16693 | 16761 | trnL1(tag) | 2.00E-06 | + |
| *Mim. nobilis* | 16764 | 16832 | trnM_0(cat) | 6.60E-07 | + |

Annotation table for *Mimachlamys senatoria*.

| Species | Start | Stop | Gene | Score | Strand |
| --- | --- | --- | --- | --- | --- |
| *Mim. senatoria* | 0 | 69 | trnN(gtt) | 6.40E-06 | + |
| *Mim. senatoria* | 79 | 141 | trnG(tcc) | 1.40E-04 | + |
| *Mim. senatoria* | 153 | 219 | trnV(tac) | 2.40E-02 | + |
| *Mim. senatoria* | 243 | 1170 | nad1_0 | 102.1 | + |
| *Mim. senatoria* | 1175 | 1242 | trnR(tcg) | 4.10E-03 | + |
| *Mim. senatoria* | 1264 | 2685 | rrnL | 0 | + |
| *Mim. senatoria* | 2856 | 4401 | cox1 | 219.9 | + |
| *Mim. senatoria* | 4438 | 4505 | trnC(gca) | 7.90E-08 | + |
| *Mim. senatoria* | 4531 | 4600 | trnA(tgc) | 2.30E-05 | + |
| *Mim. senatoria* | 4621 | 5567 | rrnS | 0 | + |
| *Mim. senatoria* | 5671 | 7372 | nad5 | 159.7 | + |
| *Mim. senatoria* | 7449 | 7725 | nad4l | 16.9 | + |
| *Mim. senatoria* | 7743 | 7896 | atp8 | 4.6 | + |
| *Mim. senatoria* | 7947 | 8018 | trnM_1(cat) | 2.00E-06 | + |
| *Mim. senatoria* | 8053 | 8557 | nad6 | 17.1 | + |
| *Mim. senatoria* | 8560 | 8625 | trnL2(taa) | 1.90E-06 | + |
| *Mim. senatoria* | 9000 | 10125 | cob | 100.5 | + |
| *Mim. senatoria* | 10075 | 10144 | trnD(gtc) | 4.90E-04 | + |
| *Mim. senatoria* | 10125 | 10912 | cox3 | 87.2 | + |
| *Mim. senatoria* | 11072 | 11140 | trnK(ttt) | 5.10E-07 | + |
| *Mim. senatoria* | 11143 | 11206 | trnF(gaa) | 1.20E-08 | + |
| *Mim. senatoria* | 11211 | 11278 | trnS2(tga) | 2.70E-03 | + |
| *Mim. senatoria* | 11284 | 11355 | trnQ(ttg) | 1.30E-07 | + |
| *Mim. senatoria* | 11362 | 11430 | trnE(ttc) | 8.10E-05 | + |
| *Mim. senatoria* | 11661 | 12315 | atp6 | 31 | + |
| *Mim. senatoria* | 12327 | 13006 | cox2 | 66.3 | + |
| *Mim. senatoria* | 13143 | 14109 | nad2 | 48.1 | + |
| *Mim. senatoria* | 14118 | 14466 | nad3 | 27.8 | + |
| *Mim. senatoria* | 14483 | 14551 | trnS1(tct) | 3.70E-03 | + |
| *Mim. senatoria* | 14557 | 15838 | nad4 | 110.7 | + |
| *Mim. senatoria* | 15840 | 15905 | trnH(gtg) | 3.90E-04 | + |
| *Mim. senatoria* | 15912 | 15980 | trnW(cca) | 1.00E-06 | + |
| *Mim. senatoria* | 15981 | 16048 | trnY(gta) | 1.10E-12 | + |
| *Mim. senatoria* | 16069 | 16134 | trnT(tgt) | 5.10E-07 | + |
| *Mim. senatoria* | 16139 | 16205 | trnP(tgg) | 5.70E-05 | + |
| *Mim. senatoria* | 16207 | 16276 | trnI(gat) | 2.90E-07 | + |
| *Mim. senatoria* | 16278 | 16344 | trnL1(tag) | 6.40E-05 | + |
| *Mim. senatoria* | 16347 | 16415 | trnM_0(cat) | 3.30E-07 | + |

Annotation table for *Amusium pleuronectes*.

| Species | Start | Stop | Gene | Score | Strand |
| --- | --- | --- | --- | --- | --- |
| *Am. pleuronectes* | 80 | 1691 | nad5 | 158 | + |
| *Am. pleuronectes* | 1828 | 2482 | atp6 | 40 | + |
| *Am. pleuronectes* | 2502 | 2569 | trnC(gca) | 1.00E-05 | + |
| *Am. pleuronectes* | 2580 | 2646 | trnY(gta) | 1.60E-13 | + |
| *Am. pleuronectes* | 2654 | 2722 | trnT(tgt) | 1.20E-05 | + |
| *Am. pleuronectes* | 4675 | 4743 | trnL1(tag) | 1.20E-07 | + |
| *Am. pleuronectes* | 4743 | 4815 | trnM(cat) | 3.10E-06 | + |
| *Am. pleuronectes* | 4838 | 4904 | trnG(tcc) | 5.70E-07 | + |
| *Am. pleuronectes* | 4912 | 4979 | trnN(gtt) | 2.00E-04 | + |
| *Am. pleuronectes* | 4980 | 5050 | trnI(gat) | 1.30E-05 | + |
| *Am. pleuronectes* | 5090 | 5159 | trnK(ttt) | 2.80E-09 | + |
| *Am. pleuronectes* | 5170 | 5235 | trnA(tgc) | 1.40E-07 | + |
| *Am. pleuronectes* | 5270 | 6239 | rrnS | 0 | + |
| *Am. pleuronectes* | 6247 | 6319 | trnQ(ttg) | 6.80E-10 | + |
| *Am. pleuronectes* | 6342 | 6410 | trnV(tac) | 5.40E-05 | + |
| *Am. pleuronectes* | 6456 | 7362 | nad1 | 105.9 | + |
| *Am. pleuronectes* | 7364 | 7434 | trnR(tcg) | 6.40E-04 | + |
| *Am. pleuronectes* | 7439 | 8859 | rrnL | 0 | + |
| *Am. pleuronectes* | 8945 | 10499 | cox1 | 221.9 | + |
| *Am. pleuronectes* | 10536 | 10600 | trnF(gaa) | 1.90E-07 | + |
| *Am. pleuronectes* | 10614 | 10680 | trnS2(tga) | 5.20E-02 | + |
| *Am. pleuronectes* | 10696 | 10762 | trnE(ttc) | 3.60E-05 | + |
| *Am. pleuronectes* | 10783 | 10918 | atp8_0 | 2.8 | + |
| *Am. pleuronectes* | 11010 | 11487 | nad6 | 16.7 | + |
| *Am. pleuronectes* | 11494 | 11557 | trnL2(taa) | 3.20E-06 | + |
| *Am. pleuronectes* | 11772 | 12867 | cob | 119 | + |
| *Am. pleuronectes* | 12903 | 13591 | cox2 | 59.5 | + |
| *Am. pleuronectes* | 13689 | 13757 | trnD(gtc) | 3.20E-05 | + |
| *Am. pleuronectes* | 13793 | 14090 | nad4l | 16.5 | + |
| *Am. pleuronectes* | 14073 | 14860 | cox3 | 85.4 | + |
| *Am. pleuronectes* | 14906 | 15890 | nad2 | 59.7 | + |
| *Am. pleuronectes* | 15901 | 16249 | nad3 | 28.7 | + |
| *Am. pleuronectes* | 16256 | 16323 | trnS1(gct) | 7.50E-03 | + |
| *Am. pleuronectes* | 16453 | 17704 | nad4 | 116.4 | + |
| *Am. pleuronectes* | 17712 | 17778 | trnH(gtg) | 9.00E-05 | + |
| *Am. pleuronectes* | 17787 | 17852 | trnW(cca) | 3.00E-05 | + |
| *Am. pleuronectes* | 17865 | 17931 | trnP(tgg) | 4.80E-08 | + |

Annotation table for *Pecten albicans*.

| Species | Start | Stop | Gene | Score | Strand |
| --- | --- | --- | --- | --- | --- |
| *Pe. albicans* | 0 | 66 | trnG(tcc) | 2.00E-06 | + |
| *Pe. albicans* | 74 | 141 | trnN(gtt) | 5.20E-03 | + |
| *Pe. albicans* | 169 | 239 | trnI(gat) | 4.60E-06 | + |
| *Pe. albicans* | 268 | 337 | trnK(ttt) | 1.30E-07 | + |
| *Pe. albicans* | 348 | 414 | trnA(tgc) | 2.30E-06 | + |
| *Pe. albicans* | 459 | 1419 | rrnS | 0 | + |
| *Pe. albicans* | 1429 | 1500 | trnQ(ttg) | 3.40E-07 | + |
| *Pe. albicans* | 1526 | 1593 | trnV(tac) | 5.20E-04 | + |
| *Pe. albicans* | 1638 | 2544 | nad1 | 105.8 | + |
| *Pe. albicans* | 2550 | 2621 | trnR(tcg) | 3.00E-03 | + |
| *Pe. albicans* | 2616 | 4071 | rrnL | 0 | + |
| *Pe. albicans* | 4153 | 5716 | cox1 | 222 | + |
| *Pe. albicans* | 5748 | 5812 | trnF(gaa) | 2.30E-07 | + |
| *Pe. albicans* | 5829 | 5895 | trnS2(tga) | 3.20E-02 | + |
| *Pe. albicans* | 5912 | 5978 | trnE(ttc) | 2.20E-04 | + |
| *Pe. albicans* | 5999 | 6134 | atp8 | 2.1 | + |
| *Pe. albicans* | 6254 | 6731 | nad6 | 16.2 | + |
| *Pe. albicans* | 6733 | 6796 | trnL2(taa) | 4.00E-06 | + |
| *Pe. albicans* | 7012 | 8110 | cob | 119.2 | + |
| *Pe. albicans* | 8223 | 8905 | cox2 | 66.5 | + |
| *Pe. albicans* | 8943 | 9011 | trnD(gtc) | 8.30E-04 | + |
| *Pe. albicans* | 9050 | 9344 | nad4l | 17.2 | + |
| *Pe. albicans* | 9327 | 10114 | cox3 | 85.8 | + |
| *Pe. albicans* | 10247 | 11162 | nad2 | 60.6 | + |
| *Pe. albicans* | 11173 | 11521 | nad3 | 28.8 | + |
| *Pe. albicans* | 11527 | 11594 | trnS1(gct) | 8.80E-02 | + |
| *Pe. albicans* | 11721 | 12972 | nad4 | 116.5 | + |
| *Pe. albicans* | 12984 | 13050 | trnH(gtg) | 4.10E-03 | + |
| *Pe. albicans* | 13060 | 13127 | trnW(cca) | 9.40E-07 | + |
| *Pe. albicans* | 13145 | 13210 | trnP(tgg) | 1.30E-07 | + |
| *Pe. albicans* | 13366 | 15013 | nad5 | 154.7 | + |
| *Pe. albicans* | 15019 | 15805 | atp6 | 0 | + |
| *Pe. albicans* | 15823 | 15889 | trnC(gca) | 1.00E-06 | + |
| *Pe. albicans* | 15899 | 15965 | trnY(gta) | 1.40E-12 | + |
| *Pe. albicans* | 15978 | 16046 | trnT(tgt) | 3.80E-07 | + |

Annotation table for *Pecten maximus*.

| Species | Start | Stop | Gene | Score | Strand |
| --- | --- | --- | --- | --- | --- |
| *Pe. maximus* | 0 | 66 | trnG(tcc) | 1.60E-06 | + |
| *Pe. maximus* | 74 | 141 | trnN(gtt) | 1.10E-04 | + |
| *Pe. maximus* | 167 | 237 | trnI(gat) | 4.80E-06 | + |
| *Pe. maximus* | 266 | 335 | trnK(ttt) | 3.40E-08 | + |
| *Pe. maximus* | 347 | 413 | trnA(tgc) | 1.70E-04 | + |
| *Pe. maximus* | 446 | 1416 | rrnS | 0 | + |
| *Pe. maximus* | 1426 | 1497 | trnQ(ttg) | 5.90E-08 | + |
| *Pe. maximus* | 1524 | 1589 | trnV(tac) | 2.50E-03 | + |
| *Pe. maximus* | 1635 | 2541 | nad1 | 105.8 | + |
| *Pe. maximus* | 2547 | 2618 | trnR(tcg) | 2.70E-03 | + |
| *Pe. maximus* | 2613 | 4066 | rrnL | 0 | + |
| *Pe. maximus* | 4148 | 5711 | cox1 | 221.1 | + |
| *Pe. maximus* | 5742 | 5806 | trnF(gaa) | 2.40E-07 | + |
| *Pe. maximus* | 5822 | 5888 | trnS2(tga) | 3.30E-02 | + |
| *Pe. maximus* | 5905 | 5971 | trnE(ttc) | 2.10E-04 | + |
| *Pe. maximus* | 5992 | 6127 | atp8 | 1.8 | + |
| *Pe. maximus* | 6238 | 6715 | nad6 | 15.7 | + |
| *Pe. maximus* | 6717 | 6780 | trnL2(taa) | 4.10E-06 | + |
| *Pe. maximus* | 6996 | 8094 | cob | 119.4 | + |
| *Pe. maximus* | 8207 | 8889 | cox2 | 66.5 | + |
| *Pe. maximus* | 8926 | 8994 | trnD(gtc) | 2.50E-03 | + |
| *Pe. maximus* | 9033 | 9327 | nad4l | 17.1 | + |
| *Pe. maximus* | 9310 | 10097 | cox3 | 84.6 | + |
| *Pe. maximus* | 10230 | 11145 | nad2 | 61.4 | + |
| *Pe. maximus* | 11156 | 11504 | nad3 | 28.7 | + |
| *Pe. maximus* | 11510 | 11577 | trnS1(gct) | 1.20E-02 | + |
| *Pe. maximus* | 11706 | 12957 | nad4 | 116.8 | + |
| *Pe. maximus* | 12969 | 13035 | trnH(gtg) | 8.60E-03 | + |
| *Pe. maximus* | 13045 | 13112 | trnW(cca) | 3.70E-07 | + |
| *Pe. maximus* | 13130 | 13195 | trnP(tgg) | 6.70E-08 | + |
| *Pe. maximus* | 13319 | 14999 | nad5 | 154.2 | + |
| *Pe. maximus* | 15005 | 15791 | atp6 | 0 | + |
| *Pe. maximus* | 15809 | 15875 | trnC(gca) | 1.40E-06 | + |
| *Pe. maximus* | 15885 | 15951 | trnY(gta) | 2.10E-14 | + |
| *Pe. maximus* | 15964 | 16032 | trnT(tgt) | 4.00E-07 | + |
| *Pe. maximus* | 17050 | 17120 | trnL1(tag) | 2.60E-07 | + |
| *Pe. maximus* | 17121 | 17190 | trnM(cat) | 8.00E-05 | + |

Annotation table for *Argopecten irradians* DQ665851, KT161259, KT161262

KU589290, NC_012977.

| Species | Start | Stop | Gene | Score | Strand |
| --- | --- | --- | --- | --- | --- |
| *Ar. irradians* | 0 | 1554 | cox1 | 224.7 | + |
| *Ar. irradians* | 1554 | 1616 | trnF(gaa) | 4.30E-07 | + |
| *Ar. irradians* | 1615 | 1684 | trnS2(tga) | 4.50E-02 | + |
| *Ar. irradians* | 1687 | 1751 | trnE(ttc) | 2.60E-04 | + |
| *Ar. irradians* | 1770 | 1905 | atp8 | 0 | + |
| *Ar. irradians* | 1952 | 2438 | nad6 | 17.4 | + |
| *Ar. irradians* | 2437 | 2501 | trnL2(taa) | 3.30E-06 | + |
| *Ar. irradians* | 2520 | 3660 | cob | 136 | + |
| *Ar. irradians* | 3684 | 4359 | cox2 | 64.9 | + |
| *Ar. irradians* | 4398 | 4692 | nad4l | 17.1 | + |
| *Ar. irradians* | 4772 | 5516 | cox3 | 82.7 | + |
| *Ar. irradians* | 5527 | 5875 | nad3 | 28.8 | + |
| *Ar. irradians* | 5874 | 5942 | trnS1(tct) | 6.50E-02 | + |
| *Ar. irradians* | 5942 | 6006 | trnN(gtt) | 4.30E-07 | + |
| *Ar. irradians* | 6007 | 7324 | nad4 | 109.5 | + |
| *Ar. irradians* | 8464 | 9394 | nad2 | 59.3 | + |
| *Ar. irradians* | 9396 | 9460 | trnD(gtc) | 1.70E-05 | + |
| *Ar. irradians* | 9461 | 9532 | trnI(gat) | 6.90E-07 | + |
| *Ar. irradians* | 9533 | 9596 | trnH(gtg) | 4.90E-03 | + |
| *Ar. irradians* | 9600 | 9666 | trnW(cca) | 4.70E-07 | + |
| *Ar. irradians* | 9666 | 9729 | trnP(tgg) | 4.30E-07 | + |
| *Ar. irradians* | 9768 | 11451 | nad5 | 159.1 | + |
| *Ar. irradians* | 11574 | 12201 | atp6 | 0 | + |
| *Ar. irradians* | 12208 | 12271 | trnC(gca) | 5.00E-06 | + |
| *Ar. irradians* | 12275 | 12339 | trnY(gta) | 6.60E-10 | + |
| *Ar. irradians* | 12338 | 12404 | trnT(tgt) | 3.20E-06 | + |
| *Ar. irradians* | 12404 | 12468 | trnA(tgc) | 1.60E-07 | + |
| *Ar. irradians* | 12466 | 12531 | trnL1(tag) | 1.60E-07 | + |
| *Ar. irradians* | 12530 | 12599 | trnM(cat) | 1.50E-07 | + |
| *Ar. irradians* | 12603 | 12663 | trnG(tcc) | 4.90E-04 | + |
| *Ar. irradians* | 12666 | 12731 | trnK(ttt) | 8.70E-09 | + |
| *Ar. irradians* | 12733 | 13642 | rrnS | 0 | + |
| *Ar. irradians* | 13639 | 13705 | trnQ(ttg) | 2.80E-10 | + |
| *Ar. irradians* | 13710 | 13773 | trnV(tac) | 1.30E-04 | + |
| *Ar. irradians* | 13771 | 14722 | nad1 | 101.8 | + |
| *Ar. irradians* | 14749 | 14816 | trnR(tcg) | 5.60E-02 | + |
| *Ar. irradians* | 14805 | 16136 | rrnL | 0 | + |

Annotation table for *Argopecten irradians* EU023915, NC_009687.

| Species | Start | Stop | Gene | Score | Strand |
| --- | --- | --- | --- | --- | --- |
| *Ar. irradians* | 1062 | 2070 | nad2 | 5.50E+01 | + |
| *Ar. irradians* | 2072 | 2136 | trnD(gtc) | 1.70E-05 | + |
| *Ar. irradians* | 2137 | 2208 | trnI(gat) | 6.80E-07 | + |
| *Ar. irradians* | 2209 | 2272 | trnH(gtg) | 4.90E-03 | + |
| *Ar. irradians* | 2276 | 2343 | trnW(cca) | 7.20E-06 | + |
| *Ar. irradians* | 2343 | 2406 | trnP(tgg) | 1.40E-06 | + |
| *Ar. irradians* | 2445 | 4134 | nad5 | 1.45E+02 | + |
| *Ar. irradians* | 4257 | 4884 | atp6 | 0 | + |
| *Ar. irradians* | 4891 | 4954 | trnC(gca) | 5.00E-06 | + |
| *Ar. irradians* | 4958 | 5022 | trnY(gta) | 6.60E-10 | + |
| *Ar. irradians* | 5021 | 5087 | trnT(tgt) | 6.70E-07 | + |
| *Ar. irradians* | 5087 | 5151 | trnA(tgc) | 1.60E-07 | + |
| *Ar. irradians* | 5149 | 5214 | trnL1(tag) | 1.60E-07 | + |
| *Ar. irradians* | 5213 | 5282 | trnM(cat) | 1.50E-07 | + |
| *Ar. irradians* | 5286 | 5346 | trnG(tcc) | 4.90E-04 | + |
| *Ar. irradians* | 5349 | 5414 | trnK(ttt) | 8.70E-09 | + |
| *Ar. irradians* | 5416 | 6325 | rrnS | 0 | + |
| *Ar. irradians* | 6322 | 6388 | trnQ(ttg) | 1.10E-09 | + |
| *Ar. irradians* | 6393 | 6456 | trnV(tac) | 4.00E-03 | + |
| *Ar. irradians* | 6454 | 7405 | nad1 | 1.00E+02 | + |
| *Ar. irradians* | 7432 | 7499 | trnR(tcg) | 5.60E-02 | + |
| *Ar. irradians* | 7490 | 8824 | rrnL | 0.00E+00 | + |
| *Ar. irradians* | 8894 | 10448 | cox1 | 224.7 | + |
| *Ar. irradians* | 10448 | 10510 | trnF(gaa) | 2.30E-04 | + |
| *Ar. irradians* | 10577 | 10799 | atp8 | 0 | + |
| *Ar. irradians* | 10846 | 11332 | nad6 | 1.03E+01 | + |
| *Ar. irradians* | 11331 | 11395 | trnL2(taa) | 3.30E-06 | + |
| *Ar. irradians* | 11414 | 12554 | cob | 1.36E+02 | + |
| *Ar. irradians* | 12578 | 13256 | cox2 | 60.1 | + |
| *Ar. irradians* | 13295 | 13589 | nad4l | 1.71E+01 | + |
| *Ar. irradians* | 13669 | 14413 | cox3 | 82.7 | + |
| *Ar. irradians* | 14424 | 14772 | nad3 | 2.86E+01 | + |
| *Ar. irradians* | 14771 | 14839 | trnS1(tct) | 6.50E-02 | + |
| *Ar. irradians* | 14839 | 14903 | trnN(gtt) | 4.30E-07 | + |
| *Ar. irradians* | 14904 | 16221 | nad4 | 1.12E+02 | + |

Annotation table for *Argopecten purpuratus*.

| Species | Start | Stop | Gene | Score | Strand |
| --- | --- | --- | --- | --- | --- |
| *Ar. purpuratus* | 0 | 1554 | cox1 | 225.2 | + |
| *Ar. purpuratus* | 1554 | 1616 | trnF(gaa) | 2.30E-08 | + |
| *Ar. purpuratus* | 1615 | 1684 | trnS2(tga) | 2.50E-02 | + |
| *Ar. purpuratus* | 1687 | 1751 | trnE(ttc) | 1.20E-04 | + |
| *Ar. purpuratus* | 1770 | 1905 | atp8_1 | 1.50E+00 | + |
| *Ar. purpuratus* | 1954 | 2440 | nad6 | 1.83E+01 | + |
| *Ar. purpuratus* | 2439 | 2503 | trnL2(taa) | 5.10E-07 | + |
| *Ar. purpuratus* | 2537 | 3662 | cob | 137 | + |
| *Ar. purpuratus* | 3686 | 4361 | cox2 | 6.48E+01 | + |
| *Ar. purpuratus* | 4409 | 4694 | nad4l | 1.79E+01 | + |
| *Ar. purpuratus* | 4774 | 5518 | cox3 | 8.26E+01 | + |
| *Ar. purpuratus* | 5530 | 5878 | nad3 | 28.1 | + |
| *Ar. purpuratus* | 5877 | 5945 | trnS1(tct) | 6.50E-02 | + |
| *Ar. purpuratus* | 5945 | 6009 | trnN(gtt) | 9.80E-06 | + |
| *Ar. purpuratus* | 6049 | 7327 | nad4 | 113.8 | + |
| *Ar. purpuratus* | 8512 | 9451 | nad2 | 57.8 | + |
| *Ar. purpuratus* | 9453 | 9517 | trnD(gtc) | 1.70E-04 | + |
| *Ar. purpuratus* | 9518 | 9589 | trnI(gat) | 9.60E-07 | + |
| *Ar. purpuratus* | 9590 | 9653 | trnH(gtg) | 5.10E-03 | + |
| *Ar. purpuratus* | 9657 | 9723 | trnW(cca) | 1.50E-07 | + |
| *Ar. purpuratus* | 9723 | 9786 | trnP(tgg) | 1.50E-05 | + |
| *Ar. purpuratus* | 9825 | 11508 | nad5 | 1.58E+02 | + |
| *Ar. purpuratus* | 11631 | 12267 | atp6 | 0 | + |
| *Ar. purpuratus* | 12266 | 12329 | trnC(gca) | 3.60E-06 | + |
| *Ar. purpuratus* | 12333 | 12397 | trnY(gta) | 1.20E-09 | + |
| *Ar. purpuratus* | 12396 | 12463 | trnT(tgt) | 1.20E-06 | + |
| *Ar. purpuratus* | 12463 | 12526 | trnA(tgc) | 9.60E-06 | + |
| *Ar. purpuratus* | 12524 | 12589 | trnL1(tag) | 8.40E-08 | + |
| *Ar. purpuratus* | 12588 | 12656 | trnM(cat) | 9.60E-07 | + |
| *Ar. purpuratus* | 12659 | 12719 | trnG(tcc) | 4.90E-04 | + |
| *Ar. purpuratus* | 12722 | 12787 | trnK(ttt) | 1.80E-08 | + |
| *Ar. purpuratus* | 12789 | 13697 | rrnS | 0.00E+00 | + |
| *Ar. purpuratus* | 13694 | 13760 | trnQ(ttg) | 6.20E-10 | + |
| *Ar. purpuratus* | 13765 | 13828 | trnV(tac) | 1.80E-04 | + |
| *Ar. purpuratus* | 13829 | 14777 | nad1 | 1.02E+02 | + |
| *Ar. purpuratus* | 14806 | 14873 | trnR(tcg) | 0 | + |
| *Ar. purpuratus* | 14865 | 16186 | rrnL | 0 | + |

Annotation table for *Argopecten ventricosus*.

| Species | Start | Stop | Gene | Score | Strand |
| --- | --- | --- | --- | --- | --- |
| *Ar. ventricosus* | 0 | 1554 | cox1 | 2.25E+02 | + |
| *Ar. ventricosus* | 1554 | 1617 | trnF(gaa) | 1.40E-08 | + |
| *Ar. ventricosus* | 1616 | 1683 | trnS2(tga) | 1.30E-03 | + |
| *Ar. ventricosus* | 1685 | 1749 | trnE(ttc) | 1.20E-04 | + |
| *Ar. ventricosus* | 1768 | 1903 | atp8 | 1.30E+00 | + |
| *Ar. ventricosus* | 1953 | 2436 | nad6 | 1.77E+01 | + |
| *Ar. ventricosus* | 2435 | 2499 | trnL2(taa) | 2.70E-07 | + |
| *Ar. ventricosus* | 2477 | 3659 | cob | 131 | + |
| *Ar. ventricosus* | 3683 | 4358 | cox2 | 6.47E+01 | + |
| *Ar. ventricosus* | 4415 | 4691 | nad4l | 18.6 | + |
| *Ar. ventricosus* | 4771 | 5515 | cox3 | 82.8 | + |
| *Ar. ventricosus* | 5524 | 5872 | nad3 | 2.84E+01 | + |
| *Ar. ventricosus* | 5871 | 5939 | trnS1(tct) | 6.40E-02 | + |
| *Ar. ventricosus* | 5939 | 6002 | trnN(gtt) | 1.20E-04 | + |
| *Ar. ventricosus* | 6003 | 7320 | nad4 | 110.7 | + |
| *Ar. ventricosus* | 8274 | 9297 | nad2 | 53 | + |
| *Ar. ventricosus* | 9301 | 9365 | trnD(gtc) | 1.10E-04 | + |
| *Ar. ventricosus* | 9366 | 9436 | trnI(gat) | 5.00E-08 | + |
| *Ar. ventricosus* | 9437 | 9500 | trnH(gtg) | 5.00E-03 | + |
| *Ar. ventricosus* | 9504 | 9568 | trnW(cca) | 7.20E-07 | + |
| *Ar. ventricosus* | 9568 | 9631 | trnP(tgg) | 1.20E-06 | + |
| *Ar. ventricosus* | 9697 | 11353 | nad5 | 1.58E+02 | + |
| *Ar. ventricosus* | 11476 | 12112 | atp6 | 0 | + |
| *Ar. ventricosus* | 12111 | 12174 | trnC(gca) | 3.60E-06 | + |
| *Ar. ventricosus* | 12176 | 12240 | trnY(gta) | 6.50E-10 | + |
| *Ar. ventricosus* | 12239 | 12306 | trnT(tgt) | 1.30E-06 | + |
| *Ar. ventricosus* | 12306 | 12369 | trnA(tgc) | 3.00E-06 | + |
| *Ar. ventricosus* | 12367 | 12433 | trnL1(tag) | 2.10E-08 | + |
| *Ar. ventricosus* | 12432 | 12499 | trnM(cat) | 1.70E-07 | + |
| *Ar. ventricosus* | 12501 | 12566 | trnG(tcc) | 2.90E-06 | + |
| *Ar. ventricosus* | 12564 | 12629 | trnK(ttt) | 9.50E-09 | + |
| *Ar. ventricosus* | 12631 | 13543 | rrnS | 0 | + |
| *Ar. ventricosus* | 13540 | 13606 | trnQ(ttg) | 1.60E-10 | + |
| *Ar. ventricosus* | 13610 | 13673 | trnV(tac) | 2.10E-04 | + |
| *Ar. ventricosus* | 13674 | 14622 | nad1 | 1.02E+02 | + |
| *Ar. ventricosus* | 14630 | 14698 | trnR(tcg) | 1.00E-02 | + |
| *Ar. ventricosus* | 14684 | 16002 | rrnL | 0 | + |

Annotation table for *Ostrea lurida*.

| Species | Start | Stop | Gene | Score | Strand |
| --- | --- | --- | --- | --- | --- |
| *O. lurida* | 0 | 1550 | cox1 | 231.8 | + |
| *O. lurida* | 1555 | 1620 | trnG(tcc) | 6.80E-12 | + |
| *O. lurida* | 1727 | 2624 | cox3 | 88.2 | + |
| *O. lurida* | 2604 | 2672 | trnI(gat) | 3.50E-09 | + |
| *O. lurida* | 2680 | 2740 | trnT(tgt) | 1.50E-04 | + |
| *O. lurida* | 2751 | 2817 | trnE(ttc) | 2.10E-07 | + |
| *O. lurida* | 2816 | 3965 | cob | 1.18E+02 | + |
| *O. lurida* | 3966 | 4659 | cox2 | 8.26E+01 | + |
| *O. lurida* | 4666 | 4730 | trnM_0(cat) | 7.00E-06 | + |
| *O. lurida* | 4737 | 4807 | trnS2(tga) | 1.40E-04 | + |
| *O. lurida* | 4825 | 4927 | atp8 | 0 | + |
| *O. lurida* | 4954 | 5018 | trnM_1(cat) | 1.90E-05 | + |
| *O. lurida* | 5077 | 5147 | trnS1(tct) | 5.50E-03 | + |
| *O. lurida* | 5149 | 5217 | trnL2(taa) | 1.70E-08 | + |
| *O. lurida* | 5219 | 5283 | trnP(tgg) | 8.70E-08 | + |
| *O. lurida* | 5264 | 5863 | rrnL_1 | 1.30E-22 | + |
| *O. lurida* | 5917 | 6904 | nad2_0 | 51.7 | + |
| *O. lurida* | 6918 | 6980 | trnC(gca) | 1.20E-07 | + |
| *O. lurida* | 6983 | 7047 | trnY(gta) | 3.40E-06 | + |
| *O. lurida* | 7071 | 7722 | atp6 | 26.9 | + |
| *O. lurida* | 7726 | 7785 | trnN(gtt) | 1.20E-06 | + |
| *O. lurida* | 7799 | 7865 | trnR(tcg) | 1.60E-05 | + |
| *O. lurida* | 7872 | 7940 | trnV(tac) | 2.10E-06 | + |
| *O. lurida* | 7961 | 8025 | trnH(gtg) | 4.30E-06 | + |
| *O. lurida* | 8046 | 9375 | nad4 | 1.23E+02 | + |
| *O. lurida* | 9383 | 10311 | rrnS | 0.00E+00 | + |
| *O. lurida* | 10359 | 11116 | rrnL_0 | 0 | + |
| *O. lurida* | 11173 | 12823 | nad5 | 1.37E+02 | + |
| *O. lurida* | 12823 | 13297 | nad6 | 9.70E+00 | + |
| *O. lurida* | 13305 | 13359 | trnQ(ttg) | 3.40E-05 | + |
| *O. lurida* | 13387 | 13726 | nad3 | 2.36E+01 | + |
| *O. lurida* | 13725 | 13789 | trnK(ttt) | 1.20E-07 | + |
| *O. lurida* | 13791 | 13855 | trnL1(tag) | 7.70E-05 | + |
| *O. lurida* | 13856 | 13921 | trnF(gaa) | 1.70E-08 | + |
| *O. lurida* | 13930 | 13994 | trnA(tgc) | 3.30E-07 | + |
| *O. lurida* | 14085 | 15018 | nad1 | 9.95E+01 | + |
| *O. lurida* | 15019 | 15301 | nad4l | 8.10E+00 | + |
| *O. lurida* | 15358 | 15422 | trnW(tca) | 5.50E-09 | + |
| *O. lurida* | 15486 | 15554 | trnD(gtc) | 1.10E-07 | + |
