# Supplementary material for: Complex mitogenomic rearrangements within the Pectinidae (Mollusca: Bivalvia)
Source: BMC Ecol Evol. 2022 Mar 10;22:29. doi: 10.1186/s12862-022-01976-0 (PMC8915466; doi:10.1186/s12862-022-01976-0)
Supplement: Supplementary file 5 — Additional file 5. Tandem repeats detected in pectinid mitogenomes. [file 12862_2022_1976_MOESM5_ESM.docx]

| Species | GenBank Accession No. | Position in genome | Period size | Copy number | Consensus size | Percent matches | Percent indels | Alignment score | A | C | G | T | Entropy (0-2) |
| --- | --- | --- | --- | --- | --- | --- | --- | --- | --- | --- | --- | --- | --- |
| *Amusium pleuronectes* | MT419374 | 1428–1457 | 15 | 2.1 | 15 | 93 | 6 | 53 | 26 | 13 | 26 | 33 | 1.93 |
|  |  | 2933–3069 | 14 | 8.9 | 15 | 75 | 24 | 85 | 10 | 76 | 0 | 13 | 1.02 |
|  |  | 2956–3065 | 26 | 4.2 | 26 | 72 | 21 | 79 | 10 | 76 | 0 | 12 | 1.02 |
|  |  | 2942–3069 | 42 | 3.3 | 40 | 75 | 21 | 128 | 10 | 77 | 0 | 12 | 1 |
|  |  | 2935–3069 | 34 | 3.9 | 33 | 81 | 14 | 141 | 10 | 77 | 0 | 12 | 1.01 |
|  |  | 2999–3061 | 12 | 5.4 | 12 | 71 | 21 | 62 | 9 | 80 | 0 | 9 | 0.89 |
|  |  | 2966–3078 | 42 | 2.6 | 45 | 82 | 13 | 146 | 9 | 76 | 0 | 13 | 1 |
|  |  | 2991–3042 | 7 | 7.4 | 7 | 75 | 16 | 54 | 9 | 80 | 0 | 9 | 0.9 |
|  |  | 3617–3644 | 11 | 2.5 | 11 | 100 | 0 | 56 | 71 | 0 | 21 | 7 | 1.09 |
|  |  | 4035–4083 | 21 | 2.3 | 22 | 86 | 6 | 66 | 12 | 67 | 0 | 20 | 1.22 |
|  |  | 4487–4539 | 22 | 2.3 | 22 | 81 | 12 | 63 | 15 | 0 | 77 | 7 | 0.98 |
| *Argopecten irradians* | DQ665851 | 8208–8246 | 11 | 3.1 | 13 | 85 | 14 | 55 | 0 | 15 | 0 | 84 | 0.62 |
|  |  | 8210–8248 | 8 | 4.9 | 8 | 84 | 12 | 53 | 0 | 10 | 0 | 89 | 0.48 |
|  | EU023915 | 575–621 | 10 | 4.5 | 10 | 86 | 5 | 58 | 63 | 19 | 14 | 2 | 1.4 |
|  |  | 838–878 | 9 | 4.8 | 9 | 82 | 11 | 50 | 0 | 14 | 0 | 85 | 0.6 |
|  | KT161259 | 8210–8248 | 8 | 4.9 | 8 | 84 | 9 | 53 | 0 | 12 | 0 | 87 | 0.55 |
|  |  | 8208–8245 | 14 | 2.6 | 14 | 91 | 4 | 58 | 0 | 15 | 0 | 84 | 0.63 |
|  | KT161262 | 8208–8246 | 11 | 3.1 | 13 | 85 | 14 | 55 | 0 | 15 | 0 | 84 | 0.62 |
|  |  | 8210–8248 | 8 | 4.9 | 8 | 84 | 12 | 53 | 0 | 10 | 0 | 89 | 0.48 |
|  | KU589290 | 8209–8247 | 11 | 3.1 | 13 | 85 | 14 | 55 | 0 | 15 | 0 | 84 | 0.62 |
|  |  | 8211–8249 | 8 | 4.9 | 8 | 84 | 12 | 53 | 0 | 10 | 0 | 89 | 0.48 |
|  | NC_009687 | 575–621 | 10 | 4.5 | 10 | 86 | 5 | 58 | 63 | 19 | 14 | 2 | 1.4 |
|  |  | 838–878 | 9 | 4.8 | 9 | 82 | 11 | 50 | 0 | 14 | 0 | 85 | 0.6 |
|  | NC_012977 | 8208–8246 | 11 | 3.1 | 13 | 85 | 14 | 55 | 0 | 15 | 0 | 84 | 0.62 |
|  |  | 8210–8248 | 8 | 4.9 | 8 | 84 | 12 | 53 | 0 | 10 | 0 | 89 | 0.48 |
| *Argopecten purpuratus* | KF601246 | 19–47 | 14 | 2.1 | 14 | 100 | 0 | 58 | 6 | 0 | 75 | 17 | 1.01 |
|  |  | 5658–5697 | 21 | 1.9 | 21 | 89 | 0 | 62 | 7 | 7 | 32 | 52 | 1.58 |
|  |  | 15699–15737 | 11 | 3.5 | 11 | 85 | 3 | 51 | 61 | 12 | 17 | 7 | 1.54 |
|  |  | 15888–15916 | 14 | 2.1 | 14 | 100 | 0 | 58 | 0 | 6 | 0 | 93 | 0.36 |
|  | KT161260 | 7936–7974 | 11 | 3.5 | 11 | 85 | 3 | 51 | 61 | 12 | 17 | 7 | 1.54 |
|  |  | 8125–8152 | 12 | 2.3 | 12 | 100 | 0 | 56 | 0 | 7 | 0 | 92 | 0.37 |
|  |  | 8525–8553 | 14 | 2.1 | 14 | 100 | 0 | 58 | 6 | 0 | 75 | 17 | 1.01 |
|  |  | 14164–14203 | 21 | 1.9 | 21 | 89 | 0 | 62 | 7 | 7 | 32 | 52 | 1.58 |
|  | KY321561 | 5620–5659 | 21 | 1.9 | 21 | 89 | 0 | 62 | 7 | 7 | 32 | 52 | 1.58 |
|  |  | 15662–15700 | 11 | 3.5 | 11 | 85 | 3 | 51 | 61 | 12 | 17 | 7 | 1.54 |
|  |  | 15849–15877 | 10 | 2.9 | 10 | 100 | 0 | 58 | 0 | 10 | 0 | 89 | 0.48 |
|  | NC_027943 | 7936–7974 | 11 | 3.5 | 11 | 85 | 3 | 51 | 61 | 12 | 17 | 7 | 1.54 |
|  |  | 8125–8152 | 12 | 2.3 | 12 | 100 | 0 | 56 | 0 | 7 | 0 | 92 | 0.37 |
|  |  | 8525–8553 | 14 | 2.1 | 14 | 100 | 0 | 58 | 6 | 0 | 75 | 17 | 1.01 |
|  |  | 14164–14203 | 21 | 1.9 | 21 | 89 | 0 | 62 | 7 | 7 | 32 | 52 | 1.58 |
| *Argopecten ventricosus* | KT161261 | 7715–7761 | 21 | 2.2 | 21 | 92 | 7 | 78 | 59 | 10 | 25 | 4 | 1.49 |
|  |  | 7714–7760 | 10 | 4.5 | 11 | 82 | 12 | 55 | 57 | 12 | 25 | 4 | 1.54 |
|  |  | 7715–7750 | 11 | 3.4 | 11 | 92 | 7 | 65 | 61 | 8 | 25 | 5 | 1.46 |
|  |  | 8371–8399 | 14 | 2.1 | 14 | 100 | 0 | 58 | 6 | 0 | 75 | 17 | 1.01 |
|  |  | 14003–14048 | 24 | 2 | 23 | 84 | 12 | 60 | 6 | 4 | 32 | 56 | 1.45 |
| *Chlamys farreri* | EF473269 | 14108–14803 | 72 | 9.7 | 72 | 99 | 0 | 1374 | 35 | 13 | 23 | 27 | 1.93 |
|  |  | 15218–15362 | 67 | 2.1 | 69 | 94 | 5 | 258 | 42 | 2 | 34 | 20 | 1.66 |
|  |  | 16707–16843 | 65 | 2.1 | 64 | 87 | 4 | 195 | 32 | 7 | 30 | 29 | 1.85 |
|  |  | 15884–17979 | 884 | 2.4 | 884 | 99 | 0 | 4156 | 33 | 9 | 24 | 32 | 1.88 |
|  |  | 17591–17727 | 65 | 2.1 | 64 | 87 | 4 | 195 | 32 | 7 | 30 | 29 | 1.85 |
|  | EU715252 | 1–154 | 65 | 2.4 | 65 | 96 | 0 | 281 | 33 | 7 | 29 | 29 | 1.85 |
|  |  | 17426–17905 | 72 | 6.7 | 72 | 98 | 0 | 924 | 35 | 15 | 23 | 25 | 1.94 |
|  |  | 18319–18462 | 68 | 2.1 | 69 | 97 | 1 | 272 | 42 | 2 | 35 | 19 | 1.66 |
|  |  | 18733–18766 | 12 | 3.1 | 11 | 88 | 12 | 52 | 17 | 0 | 64 | 17 | 1.29 |
|  |  | 19760–19896 | 65 | 2.1 | 64 | 86 | 4 | 186 | 31 | 8 | 30 | 29 | 1.86 |
|  |  | 18942–20775 | 880 | 2.1 | 879 | 98 | 0 | 3564 | 32 | 9 | 24 | 32 | 1.89 |
|  |  | 20640–20904 | 64 | 4.1 | 64 | 95 | 1 | 424 | 30 | 8 | 30 | 29 | 1.87 |
|  |  | 19760–21695 | 1008 | 1.9 | 1008 | 99 | 0 | 3809 | 32 | 9 | 25 | 32 | 1.89 |
|  | NC_012138 | 1–154 | 65 | 2.4 | 65 | 96 | 0 | 281 | 33 | 7 | 29 | 29 | 1.85 |
|  |  | 17426–17905 | 72 | 6.7 | 72 | 98 | 0 | 924 | 35 | 15 | 23 | 25 | 1.94 |
|  |  | 18319–18462 | 68 | 2.1 | 69 | 97 | 1 | 272 | 42 | 2 | 35 | 19 | 1.66 |
|  |  | 18733–18766 | 12 | 3.1 | 11 | 88 | 12 | 52 | 17 | 0 | 64 | 17 | 1.29 |
|  |  | 19760–19896 | 65 | 2.1 | 64 | 86 | 4 | 186 | 31 | 8 | 30 | 29 | 1.86 |
|  |  | 18942–20775 | 880 | 2.1 | 879 | 98 | 0 | 3564 | 32 | 9 | 24 | 32 | 1.89 |
|  |  | 20640–20904 | 64 | 4.1 | 64 | 95 | 1 | 424 | 30 | 8 | 30 | 29 | 1.87 |
|  |  | 19760–21695 | 1008 | 1.9 | 1008 | 99 | 0 | 3809 | 32 | 9 | 25 | 32 | 1.89 |
| *Crassadoma gigantea* | MH016739 | 5664–5707 | 12 | 3.7 | 12 | 100 | 0 | 88 | 22 | 36 | 0 | 40 | 1.54 |
| *Mimachlamys nobilis* | FJ415225 | 31–74 | 22 | 2 | 22 | 82 | 13 | 54 | 18 | 6 | 9 | 65 | 1.42 |
|  |  | 17562–17588 | 12 | 2.3 | 12 | 100 | 0 | 54 | 14 | 0 | 85 | 0 | 0.61 |
|  |  | 17794–17883 | 44 | 2 | 44 | 89 | 8 | 137 | 13 | 20 | 42 | 24 | 1.87 |
|  | FJ595958 | 17507–17596 | 44 | 2 | 44 | 89 | 8 | 137 | 13 | 20 | 42 | 24 | 1.87 |
|  |  | 17707–17750 | 22 | 2 | 22 | 82 | 13 | 54 | 18 | 6 | 9 | 65 | 1.42 |
|  | NC_011608 | 31–74 | 22 | 2 | 22 | 82 | 13 | 54 | 18 | 6 | 9 | 65 | 1.42 |
|  |  | 17562–17588 | 12 | 2.3 | 12 | 100 | 0 | 54 | 14 | 0 | 85 | 0 | 0.61 |
|  |  | 17794–17883 | 44 | 2 | 44 | 89 | 8 | 137 | 13 | 20 | 42 | 24 | 1.87 |
| *Placopecten magellanicus* | DQ088274 | 2290–2842 | 79 | 7 | 79 | 100 | 0 | 1106 | 21 | 15 | 25 | 37 | 1.92 |
|  |  | 19733–19759 | 10 | 2.7 | 10 | 100 | 0 | 54 | 85 | 14 | 0 | 0 | 0.61 |
|  |  | 21168–21194 | 10 | 2.7 | 10 | 100 | 0 | 54 | 85 | 14 | 0 | 0 | 0.61 |
|  |  | 19088–21957 | 1435 | 2 | 1435 | 100 | 0 | 5740 | 31 | 12 | 27 | 27 | 1.93 |
|  |  | 21971–22007 | 11 | 3.4 | 11 | 100 | 0 | 74 | 0 | 0 | 16 | 83 | 0.64 |
|  | NC_007234 | 2290–2842 | 79 | 7 | 79 | 100 | 0 | 1106 | 21 | 15 | 25 | 37 | 1.92 |
|  |  | 19733–19759 | 10 | 2.7 | 10 | 100 | 0 | 54 | 85 | 14 | 0 | 0 | 0.61 |
|  |  | 21168–21194 | 10 | 2.7 | 10 | 100 | 0 | 54 | 85 | 14 | 0 | 0 | 0.61 |
|  |  | 19088–21957 | 1435 | 2 | 1435 | 100 | 0 | 5740 | 31 | 12 | 27 | 27 | 1.93 |
|  |  | 21971–22007 | 11 | 3.4 | 11 | 100 | 0 | 74 | 0 | 0 | 16 | 83 | 0.64 |
