# Supplementary material for: Complex mitogenomic rearrangements within the Pectinidae (Mollusca: Bivalvia)
Source: BMC Ecol Evol. 2022 Mar 10;22:29. doi: 10.1186/s12862-022-01976-0 (PMC8915466; doi:10.1186/s12862-022-01976-0)
Supplement: Supplementary file 6 — Additional file 6. Partitioning and substitution model information for all eight datasets used in the Maximum Likelihood analyses. [file 12862_2022_1976_MOESM6_ESM.docx]

**Dataset A: all PCG and rRNA genes treated with Gblocks.**

#nexus

begin sets;

charset rrnS_rrnL = 1-895 896-2107;

charset cob_cox1_cox3_nad1_nad4 = 2108-3163 3164-4714 5378-6115 6116-7024 8295-9536;

charset cox2_nad2_nad3_nad4l_nad5_nad6_atp6_atp8 = 4715-5377 7025-7948 7949-8294 9537-9803 9804-11289 11290-11712 11712-12273 12274-12417;

charpartition mymodels =

GTR+F+G4: rrnS_rrnL,

GTR+F+I+G4: cob_cox1_cox3_nad1_nad4,

TVM+F+I+G4: cox2_nad2_nad3_nad4l_nad5_nad6_atp6_atp8;

end;

**Dataset B: all PCG amino acid sequences and rRNA genes treated with Gblocks.**

#nexus

begin sets;

charset rrnS_rrnL = 1-895 896-2107;

charset cob_cox3_nad1_nad4_nad4l = 1-352 1091-1336 1337-1639 2063-2476 2477-2565;

charset cox1 = 353-869;

charset cox2_nad2_nad3_nad5_nad6_atp6_atp8 = 870-1090 1640-1947 1948-2062 2566-3060 3061-3201 3202-3388 3389-3436;

charpartition mymodels =

GTR+F+G4: rrnS_rrnL,

mtZOA+F+G4: cob_cox3_nad1_nad4_nad4l,

mtZOA+G4: cox1,

mtInv+F+G4: cox2_nad2_nad3_nad5_nad6_atp6_atp8;

end;

**Dataset C: all PCGs.**

#nexus

begin sets;

charset cob_cox1_cox3_nad1_nad3_nad4 = 1-1560 1561-3339 4246-5211 5212-6207 7378-7812 7813-9222;

charset cox2_nad2_nad4l_nad5_nad6_atp6_atp8 = 3340-4245 6208-7377 9223-9534 9535-11667 11668-12192 12193-13149 13150-13343;

charpartition mymodels =

GTR+F+I+G4: cob_cox1_cox3_nad1_nad3_nad4,

TVM+F+I+G4: cox2_nad2_nad4l_nad5_nad6_atp6_atp8;

end;

**Dataset D: all PCGs treated with Gblocks.**

#nexus

begin sets;

charset cob_cox1_cox3_nad1_nad4 = 1-1056 1057-2607 3271-4008 4009-4917 6187-7428;

charset cox2_nad2_nad3_nad4l_nad5_nad6_atp6_atp8 = 2608-3270 4918-5841 5842-6186 7429-7695 7696-9179 9180-9602 9603-10163 10164-10307;

charpartition mymodels =

GTR+F+I+G4: cob_cox1_cox3_nad1_nad4,

TVM+F+I+G4: cox2_nad2_nad3_nad4l_nad5_nad6_atp6_atp8;

end;

**Dataset E: all PCG amino acid sequences.**

#nexus

begin sets;

charset cob_cox1_cox2_cox3_nad1_nad3_nad4_nad4l = 1-519 520-1111 1112-1413 1414-1734 1735-2065 2455-2598 2599-3067 3068-3170;

charset nad2_nad5_nad6_atp6_atp8 = 2066-2454 3171-3879 3880-4053 4054-4371 4372-4431;

charpartition mymodels =

mtInv+F+R4: cob_cox1_cox2_cox3_nad1_nad3_nad4_nad4l,

mtInv+F+G4: nad2_nad5_nad6_atp6_atp8;

end;

**Dataset F: all PCG amino acid sequences treated with Gblocks.**

#nexus

begin sets;

charset cob_cox1_cox2_cox3_nad1_nad3_nad4_nad4l = 1-352 353-869 870-1090 1091-1336 1337-1639 1948-2062 2063-2476 2477-2565;

charset nad2_nad5_nad6_atp6_atp8 = 1640-1947 2566-3060 3061-3201 3202-3388 3389-3436;

charpartition mymodels =

mtInv+F+R3: cob_cox1_cox2_cox3_nad1_nad3_nad4_nad4l,

mtInv+F+G4: nad2_nad5_nad6_atp6_atp8;

end;

**Dataset G: rRNA genes.**

#nexus

begin sets;

charset rrnS = 1-1028;

charset rrnL = 1029-2601;

charpartition mymodels =

GTR+F+G4: rrnS,

TPM3+F+I+G4: rrnL;

end;

**Dataset H: rRNA genes treated with Gblocks.**

#nexus

begin sets;

charset rrnS_rrnL = 1-895 896-2107;

charpartition mymodels =

GTR+F+G4: rrnS_rrnL;

end;
