# Supplementary material for: Complex mitogenomic rearrangements within the Pectinidae (Mollusca: Bivalvia)
Source: BMC Ecol Evol. 2022 Mar 10;22:29. doi: 10.1186/s12862-022-01976-0 (PMC8915466; doi:10.1186/s12862-022-01976-0)
Supplement: Supplementary file 7 — Additional file 7. Partitioning schemes and prior model parameter settings for all eight datasets used in the Bayesian analyses. [file 12862_2022_1976_MOESM7_ESM.docx]

**Dataset A: all PCG and rRNA genes treated with Gblocks.**

BEGIN mrbayes;

charset Subset1 = 896-2107 1-895;

charset Subset2 = 3164-4714 6116-7024 2108-3163 7949-8294 8295-9536 5378-6115;

charset Subset3 = 4715-5377 11713-12273 9537-9803 9804-11289 7025-7948 12274-12417 11290-11712;

partition PartitionFinder = 3:Subset1, Subset2, Subset3;

set partition=PartitionFinder;

lset applyto=(1) nst=6 rates=gamma;

lset applyto=(2) nst=6 rates=invgamma;

lset applyto=(3) nst=6 rates=invgamma;

prset applyto=(all) ratepr=variable;

unlink statefreq=(all) revmat=(all) shape=(all) pinvar=(all) tratio=(all);

set autoclose=yes nowarnings=yes;

mcmc ngen=1500000 nruns=2 nchains=4 temp=0.100 samplefreq=500 mcmcdiagn=yes relburnin=yes burninfrac=0.25 stoprule=no starttree=random Savebrlens=yes Ordertaxa=yes;

sump burninfrac=0.25 nruns=2 outputname=sumpoutput.out;

sumt burninfrac=0.25 nruns=2 ntrees=1 minpartfreq=0.05 contype=Halfcompat conformat=Figtree;

END;

**Dataset B: all PCG amino acid sequences and rRNA genes treated with Gblocks.**

BEGIN mrbayes;

charset Subset1 = 1-895 896-2107;

charset Subset2 = 4170-4583 3444-3746 2108-2459;

charset Subset3 = 2460-2976;

charset Subset4 = 4584-4672 2977-3197 4055-4169;

charset Subset5 = 3198-3443;

charset Subset6 = 5168-5308 3747-4054;

charset Subset7 = 4673-5167;

charset Subset8 = 5309-5495 5496-5543;

partition PartitionFinder = 8:Subset1, Subset2, Subset3, Subset4, Subset5, Subset6, Subset7, Subset8;

set partition=PartitionFinder;

lset applyto=(1) nst=6 rates=gamma;

lset applyto=(2) rates=gamma;

prset applyto=(2) aamodelpr=fixed(cprev);

lset applyto=(3) rates=gamma;

prset applyto=(3) aamodelpr=fixed(wag);

lset applyto=(4) rates=gamma;

prset applyto=(4) aamodelpr=fixed(wag);

lset applyto=(5) rates=gamma;

prset applyto=(5) aamodelpr=fixed(wag);

lset applyto=(6) rates=gamma;

prset applyto=(6) aamodelpr=fixed(wag);

lset applyto=(7) rates=gamma;

prset applyto=(7) aamodelpr=fixed(wag);

lset applyto=(8) rates=gamma;

prset applyto=(8) aamodelpr=fixed(wag);

prset applyto=(all) ratepr=variable;

unlink statefreq=(all) revmat=(all) shape=(all) pinvar=(all) tratio=(all);

set autoclose=yes nowarnings=yes;

mcmc append=yes ngen=1500000 nruns=2 nchains=4 temp=0.100 samplefreq=500 mcmcdiagn=yes relburnin=yes burninfrac=0.25 stoprule=no starttree=random Savebrlens=yes Ordertaxa=yes;

sump burninfrac=0.25 nruns=2 outputname=sumpoutput.out;

sumt burninfrac=0.25 nruns=2 ntrees=1 minpartfreq=0.05 contype=Halfcompat conformat=Figtree;

END;

**Dataset C: all PCGs.**

BEGIN mrbayes;

charset Subset1 = 5212-6207 1-1560 7378-7812 4246-5211 7813-9222;

charset Subset2 = 1561-3339;

charset Subset3 = 13150-13343 3340-4245 9223-9534 11668-12192 12193-13149 9535-11667 6208-7377;

partition PartitionFinder = 3:Subset1, Subset2, Subset3;

set partition=PartitionFinder;

lset applyto=(1) nst=6 rates=gamma;

lset applyto=(2) nst=2 rates=invgamma;

lset applyto=(3) nst=6 rates=invgamma;

prset applyto=(all) ratepr=variable;

unlink statefreq=(all) revmat=(all) shape=(all) pinvar=(all) tratio=(all);

set autoclose=yes nowarnings=yes;

mcmc ngen=1500000 nruns=2 nchains=4 temp=0.100 samplefreq=500 mcmcdiagn=yes relburnin=yes burninfrac=0.25 stoprule=no starttree=random Savebrlens=yes Ordertaxa=yes;

sump burninfrac=0.25 nruns=2 outputname=sumpoutput.out;

sumt burninfrac=0.25 nruns=2 ntrees=1 minpartfreq=0.05 contype=Halfcompat conformat=Figtree;

END;

**Dataset D: all PCGs treated with Gblocks.**

BEGIN mrbayes;

charset Subset1 = 1057-2607 4009-4917 1-1056 6187-7428 3271-4008;

charset Subset2 = 2608-3270 9603-10163 5842-6186 7429-7695 4918-5841 7696-9179 10164-10307 9180-9602;

partition PartitionFinder = 2:Subset1, Subset2;

set partition=PartitionFinder;

lset applyto=(1) nst=6 rates=invgamma;

lset applyto=(2) nst=6 rates=invgamma;

prset applyto=(all) ratepr=variable;

unlink statefreq=(all) revmat=(all) shape=(all) pinvar=(all) tratio=(all);

set autoclose=yes nowarnings=yes;

mcmc ngen=1500000 nruns=2 nchains=4 temp=0.100 samplefreq=500 mcmcdiagn=yes relburnin=yes burninfrac=0.25 stoprule=no starttree=random Savebrlens=yes Ordertaxa=yes;

sump burninfrac=0.25 nruns=2 outputname=sumpoutput.out;

sumt burninfrac=0.25 nruns=2 ntrees=1 minpartfreq=0.05 contype=Halfcompat conformat=Figtree;

END;

**Dataset E: all PCG amino acid sequences.**

BEGIN mrbayes;

charset Subset1 = 1-519;

charset Subset2 = 520-1111;

charset Subset3 = 1414-1734 1112-1413 2455-2598 3068-3170;

charset Subset4 = 2599-3067 1735-2065;

charset Subset5 = 4054-4371 2066-2454;

charset Subset6 = 3171-3879 3880-4053;

charset Subset7 = 4372-4431;

partition PartitionFinder = 7:Subset1, Subset2, Subset3, Subset4, Subset5, Subset6, Subset7;

set partition=PartitionFinder;

lset applyto=(1) rates=gamma;

prset applyto=(1) aamodelpr=fixed(wag);

lset applyto=(2) rates=gamma;

prset applyto=(2) aamodelpr=fixed(wag);

lset applyto=(3) rates=gamma;

prset applyto=(3) aamodelpr=fixed(wag);

lset applyto=(4) rates=gamma;

prset applyto=(4) aamodelpr=fixed(cprev);

lset applyto=(5) rates=gamma;

prset applyto=(5) aamodelpr=fixed(wag);

lset applyto=(6) rates=gamma;

prset applyto=(6) aamodelpr=fixed(wag);

lset applyto=(7) rates=propinv;

prset applyto=(7) aamodelpr=fixed(vt);

prset applyto=(all) ratepr=variable;

unlink statefreq=(all) revmat=(all) shape=(all) pinvar=(all) tratio=(all);

set autoclose=yes nowarnings=yes;

mcmc append=yes ngen=1500000 nruns=2 nchains=4 temp=0.100 samplefreq=500 mcmcdiagn=yes relburnin=yes burninfrac=0.25 stoprule=no starttree=random Savebrlens=yes Ordertaxa=yes;

sump burninfrac=0.25 nruns=2 outputname=sumpoutput.out;

sumt burninfrac=0.25 nruns=2 ntrees=1 minpartfreq=0.05 contype=Halfcompat conformat=Figtree;

END;

**Dataset F: all PCG amino acid sequences treated with Gblocks.**

BEGIN mrbayes;

charset Subset1 = 2063-2476 1337-1639 1-352;

charset Subset2 = 353-869;

charset Subset3 = 2477-2565 870-1090 1948-2062;

charset Subset4 = 1091-1336;

charset Subset5 = 3061-3201 1640-1947;

charset Subset6 = 2566-3060;

charset Subset7 = 3202-3388 3389-3436;

partition PartitionFinder = 7:Subset1, Subset2, Subset3, Subset4, Subset5, Subset6, Subset7;

set partition=PartitionFinder;

lset applyto=(1) rates=gamma;

prset applyto=(1) aamodelpr=fixed(cprev);

lset applyto=(2) rates=gamma;

prset applyto=(2) aamodelpr=fixed(wag);

lset applyto=(3) rates=gamma;

prset applyto=(3) aamodelpr=fixed(wag);

lset applyto=(4) rates=gamma;

prset applyto=(4) aamodelpr=fixed(wag);

lset applyto=(5) rates=gamma;

prset applyto=(5) aamodelpr=fixed(wag);

lset applyto=(6) rates=gamma;

prset applyto=(6) aamodelpr=fixed(wag);

lset applyto=(7) rates=gamma;

prset applyto=(7) aamodelpr=fixed(wag);

prset applyto=(all) ratepr=variable;

unlink statefreq=(all) revmat=(all) shape=(all) pinvar=(all) tratio=(all);

set autoclose=yes nowarnings=yes;

mcmc append=yes ngen=1500000 nruns=2 nchains=4 temp=0.100 samplefreq=500 mcmcdiagn=yes relburnin=yes burninfrac=0.25 stoprule=no starttree=random Savebrlens=yes Ordertaxa=yes;

sump burninfrac=0.25 nruns=2 outputname=sumpoutput.out;

sumt burninfrac=0.25 nruns=2 ntrees=1 minpartfreq=0.05 contype=Halfcompat conformat=Figtree;

END;

**Dataset G: rRNA genes.**

BEGIN mrbayes;

charset Subset1 = 1-1028;

charset Subset2 = 1029-2601;

partition PartitionFinder = 2:Subset1, Subset2;

set partition=PartitionFinder;

lset applyto=(1) nst=6 rates=gamma;

lset applyto=(2) nst=6 rates=invgamma;

prset applyto=(all) ratepr=variable;

unlink statefreq=(all) revmat=(all) shape=(all) pinvar=(all) tratio=(all);

set autoclose=yes nowarnings=yes;

mcmc ngen=1500000 nruns=2 nchains=4 temp=0.100 samplefreq=500 mcmcdiagn=yes relburnin=yes burninfrac=0.25 stoprule=no starttree=random Savebrlens=yes Ordertaxa=yes;

sump burninfrac=0.25 nruns=2 outputname=sumpoutput.out;

sumt burninfrac=0.25 nruns=2 ntrees=1 minpartfreq=0.05 contype=Halfcompat conformat=Figtree;

END;

**Dataset H: rRNA genes treated with Gblocks.**

BEGIN mrbayes;

charset Subset1 = 1-895 896-2107;

partition PartitionFinder = 1:Subset1;

set partition=PartitionFinder;

lset applyto=(1) nst=6 rates=gamma;

set autoclose=yes nowarnings=yes;

mcmc ngen=1500000 nruns=2 nchains=4 temp=0.100 samplefreq=500 mcmcdiagn=yes relburnin=yes burninfrac=0.25 stoprule=no starttree=random Savebrlens=yes Ordertaxa=yes;

sump burninfrac=0.25 nruns=2 outputname=sumpoutput.out;

sumt burninfrac=0.25 nruns=2 ntrees=1 minpartfreq=0.05 contype=Halfcompat conformat=Figtree;

END;
